# Plant miR8126-3p and miR8126-5p Decrease Lipid Accumulation through Modulation of Metabolic Genes in a Human Hepatocyte Model That Mimics Steatosis

**DOI:** 10.3390/ijms25031721

**Published:** 2024-01-31

**Authors:** Ester Díez-Sainz, Paula Aranaz, Ez-Zoubir Amri, José I. Riezu-Boj, Silvia Lorente-Cebrián, Fermín I. Milagro

**Affiliations:** 1 Department of Nutrition, Food Science and Physiology, and Center for Nutrition Research, Faculty of Pharmacy and Nutrition, University of Navarra, 31008 Pamplona, Spain; ediezsainz@alumni.unav.es (E.D.-S.); paranaz@unav.es (P.A.); jiriezu@unav.es (J.I.R.-B.); fmilagro@unav.es (F.I.M.); 2 Navarra Institute for Health Research (IdiSNA), 31008 Pamplona, Spain; 3 CNRS, Inserm, iBV, Université Côte d’Azur, 06107 Nice, France; amri@unice.fr; 4 Department of Pharmacology, Physiology and Legal and Forensic Medicine, Faculty of Health and Sport Science, University of Zaragoza, 50009 Zaragoza, Spain; 5 Instituto Agroalimentario de Aragón-IA2, Universidad de Zaragoza-Centro de Investigación y Tecnología Agroalimentaria (CITA), 50013 Zaragoza, Spain; 6 Aragón Health Research Institute (IIS-Aragon), 50009 Zaragoza, Spain; 7 Centro de Investigación Biomédica en Red Fisiopatología de la Obesidad y Nutrición (CIBERobn), Instituto de Salud Carlos III, 28029 Madrid, Spain

**Keywords:** plant microRNA, cross-kingdom regulation, metabolism, fatty liver, NAFLD, steatosis

## Abstract

Plant-based food interventions are promising therapeutic approaches for non-alcoholic fatty liver disease (NAFLD) treatment, and microRNAs (miRNAs) have emerged as functional bioactive components of dietary plants involved in cross-kingdom communication. Deeper investigations are needed to determine the potential impact of plant miRNAs in NAFLD. This study aimed to identify plant miRNAs that could eventually modulate the expression of human metabolic genes and protect against the progression of hepatic steatosis. Plant miRNAs from the miRBase were used to predict human target genes, and miR8126-3p and miR8126-5p were selected as candidates for their potential role in inhibiting glucose and lipid metabolism-related genes. Human HepG2 cells were transfected with plant miRNA mimics and then exposed to a mixture of oleic and palmitic acids to mimic steatosis. miR8126-3p and miR8126-5p transfections inhibited the expression of the putative target genes *QKI* and *MAPKAPK2*, respectively, and had an impact on the expression profile of key metabolic genes, including *PPARA* and *SREBF1*. Quantification of intrahepatic triglycerides revealed that miR8126-3p and miR8126-5p attenuated lipid accumulation. These findings suggest that plant miR8126-3p and miR8126-5p would induce metabolic changes in human hepatocytes eventually protecting against lipid accumulation, and thus, they could be potential therapeutic tools for preventing and alleviating lipid accumulation.

## 1. Introduction

Non-alcoholic fatty liver disease (NAFLD) is one of the most common liver-associated diseases and is characterized by excessive lipid accumulation in hepatocytes [1]. The NAFLD incidence rate is rapidly increasing worldwide, being estimated at approximately 30%; thus, it is considered an emerging epidemic [2]. Several factors could contribute to NAFLD development, including dyslipidemia, genetic susceptibility, diet and lifestyle patterns, gut microbiota dysbiosis, and metabolic diseases [3,4]. The disturbance of lipid metabolism homeostasis in the liver induces an increase in intrahepatic triglyceride content leading to liver steatosis [1]. NAFLD comprises a broad spectrum of diseases since simple steatosis (non-alcoholic fatty liver, NAFL) can progress to non-alcoholic steatohepatitis (NASH), which could potentially lead to liver cirrhosis and eventually hepatocellular carcinoma (HCC) [5]. Progression to the most severe stages of the diseases (cirrhosis and HCC) is irreversible, raising liver failure and mortality risk [6]. Therefore, therapeutic strategies aimed at controlling the early stages of the disease, such as hepatic lipid accumulation, could be crucial to prevent and/or reverse NAFLD [7].

Treatment of NAFLD is currently challenging. Nowadays, the Food and Drug Administration (FDA) has not approved any therapy yet, underscoring the urgent need to identify therapeutic targets for the effective management of the disease. Dietary intervention is considered the main approach for NAFLD management [8]. It has been extensively documented that nutrition and dietary patterns can have an impact on NAFLD pathophysiology [9,10]. For instance, plant-rich diets like the Mediterranean diet could be linked with NAFLD prevention and/or reversion [11,12]. In this context, compelling evidence demonstrates the therapeutic potential of a wide range of plants and plant-derived foods against NAFLD (i.e., fruits, vegetables, olive oil, grains, legumes, and herbal plants), as these plants and plant-derived foods could decrease lipid accumulation, oxidative stress, and inflammation and modulate the gut microbiota [13,14,15]. The beneficial effects of plants preventing and/or alleviating NAFLD have been attributed to several bioactive compounds, including polyphenols, omega-3 fatty acids, carotenoids, phytosterols, terpenoids, and polysaccharides [13,14,15]. Notably, emerging reports have suggested that microRNAs (miRNAs) could be a new class of bioactive plant compounds with crucial biological roles in mammals [16,17].

miRNAs are non-coding RNAs of about 22 nucleotides in length that act as post-transcriptional gene expression regulators [18]. miRNAs exert a key control over protein synthesis, through the base-complementary binding to messenger RNAs (mRNAs), which either promote mRNA degradation or repress translation [18]. Plant miRNAs play a critical role at the intra-kingdom level, maintaining the homeostasis of several biological processes, such as growth and development, stress responses, and metabolism [19,20,21]. Significant evidence supports a key involvement of plant miRNAs in cross-kingdom communication, shaping bacteria and eukaryote gene expression. Plant miRNAs’ cross-kingdom functions include the regulation of immune system responses, the gut microbiota, metabolism, and viral infections [17]. The bioavailability of plant exogenous miRNAs (exomiRs) in animals, including humans, could be achieved through diet, as they have been detected in the intestine, circulatory system, and peripheral tissues [22,23]. However, controversial findings have been found with disparity in results [24]. For instance, plant miR168a was detected in human and animal serum and organs and inhibited the lipoprotein receptor adapter protein 1 (*LDLRAP1*) in mouse livers, increasing plasma LDL cholesterol; other authors were not able to reproduce these results [25,26]. Notably, conclusive evidence has demonstrated that the plant miRNA profile in a host is determined by dietary patterns [27]. Hence, exomiRs stand out as a novel bioactive molecule and a crucial underlying factor of the effects promoted by plant-based diets on human health [28]. ExomiRs could be a promising therapeutic target for the management of several diseases, including metabolic and cardiovascular diseases [29]. However, further research is needed to unveil the exact mechanism of action underpinning the cross-kingdom biological functions of plant miRNAs and to elucidate their therapeutic potential in humans.

The aim of the present study was to investigate the role of plant miRNAs in energy metabolism and lipid accumulation in an in vitro model of hepatic steatosis.

## 2. Results

### 2.1. Identification of Human Putative Target Genes of Plant miR8126-3p and miR8126-5p

*Prunus persica* was used to select plant miRNAs due to its well-annotated miRNA profile and because it represents an edible fruit widely consumed at the global level (https://mirbase.org/results/?query=prunus+persica) (https://plants.ensembl.org/Prunus_persica/Info/Index) (accessed on 12 April 2022). Therefore, 214 miRNAs belonging to *Prunus Persica* genome were selected from miRBase to predict human putative target genes. TAPIR and psRNATarget [30,31] prediction tools were used to identify potential human target genes of plant miRNAs, as their algorithms were specifically designed for the prediction of plant miRNA targets. Only miRNA sequences with putative target genes common to TAPIR and psRNATarget (scoring schema V1 and V2) were selected for further studies in order to potentially choose the most accurate predictions [32]: 163 miRNA–mRNA pairs were common outputs, comprising 91 different miRNAs and 112 different transcripts (from 100 different genes). Finally, a bibliographic search was performed, with the 100 genes common to the prediction algorithm, to select target genes related to lipid metabolism, liver metabolism, and/or NAFLD. Applying these criteria, miR8126-3p and miR8126-5p (identified in *Prunus Persica*) and the putative target genes *QKI* (Quaking Homolog, KH Domain RNA Binding) and *MAPKAPK2* (MAPK Activated Protein Kinase 2), respectively, were selected for subsequent in vitro studies.

Prediction algorithms identified 11 (psRNATarget scoring schema V1), 24 (psRNATarget scoring schema V2), and 6 (TAPIR) targets of plant miR8126-3p (Table 1). A total of 24 different transcripts (19 different genes) were identified as putative targets of plant miR8126-3p since there were transcripts that appeared in more than one of the algorithms and transcripts that were encoded by the same gene. Gene Ontology (GO) analyses were conducted to search enrichment pathways of the set of predicted targets consisting of 19 genes (*FHAD1*, *INTS8*, *CAPSL*, *CASC3*, *ADAMTS18*, *RBM47*, *QKI*, *TMEM255A*, *ANKRD7*, *KIAA1644*, *MAOA*, *LATS1*, *RPL37*, *SIDT2*, *TTPAL*, *IYD*, *ATP1B4*, *RORA*, *NCOR1*), of which GeneCodis reported 5 (*ANKRD7*, *CAPSL*, *FHAD1*, *SHISAL1*, and *TTPAL*) as unannotated inputs. Enrichment pathways included processes such as RNA splicing (*CASC3* and *QKI*), thyroid hormone metabolic process (*IYD*), and regulation of glucose metabolic process (*RORA*) (Supplementary Table S1). Nevertheless, the only common targets of the three bioinformatic servers (psRNATarget scoring schema V1 and V2, and TAPIR) were the transcripts NM_006775, NM_206853, NM_206854, NM_206855, which code for the *QKI* (Quaking Homolog, KH Domain RNA Binding) gene, and the transcripts NM_052929 and NM_001042625, which code for *FHAD1* (Forkhead Associated Phosphopeptide Binding Domain 1) and *CAPSL* (Calcyphosine Like) genes, respectively (Table 1 and Supplementary Figure S1A). Providing that *QKI* has been previously associated with cholesterol biosynthesis, lipid metabolism, liver triglycerides, and hepatic steatosis [33,34,35], we aimed to evaluate the influence of plant miR8126-3p on target genes and lipid metabolism in human hepatocyte HepG2 cell model. FHAD1 (https://www.proteinatlas.org/ENSG00000142621-FHAD1/subcellular; last accessed on 13 July 2023) and CAPSL (https://www.proteinatlas.org/ENSG00000152611-CAPSL/subcellular; last accessed on 13 July 2023) protein levels were negligible in HepG2; thus, only *QKI* expression was assessed in this human hepatocyte in vitro model.

Bioinformatic predictions reported 5 (psRNATarget scoring schema V1), 20 (psRNATarget scoring schema V2), and 2 (TAPIR) putative targets of plant miR8126-5p (Table 2). Altogether, 21 different transcripts (corresponding to 17 genes) were predicted as potential targets of miR8126-5p because there were transcripts that appeared in more than one of the algorithms and transcripts that were encoded by the same gene. Subsequently, GO analyses were performed with the 17 predicted target genes (*MAPKAPK2, KLHL4*, *EOGT*, *PPP1R13L*, *FNDC9*, *KLHL8*, *GNS*, *NEDD4L*, *TBC1D15*, *RNF14*, *TMEM135*, *MBNL2*, *HSPE1-MOB4*, *GPATCH8*, *ANP32E*, *CXorf40A*, *SI*), of which GeneCodis reported the *FNDC9* gene as an unannotated input (Supplementary Table S2). A wide range of enriched pathways was reported, including the p38MAPK cascade (*MAPKAPK2*), sucrose catabolic process (*SI*), and response to food (*TMEM135*). Nevertheless, the transcripts NM_032960 and NM_004759, which code for *MAPKAPK2* (MAPK Activated Protein Kinase 2, also named MK2), were the only targets common to the three prediction algorithms (Table 2 and Supplementary Figure S1B). Interestingly, *MAPKAPK2* has been previously reported to be involved in lipid metabolism [36,37,38], and its expression has been also reported in HepG2 (https://www.proteinatlas.org/ENSG00000162889-MAPKAPK2/subcellular; last accessed on 13 July 2023).

The evidence collected from the above-presented bioinformatic analyses and literature search led to the selection of miR8126-3p and miR8126-5p and putative target genes *QKI* and *MAPKAPK2*, respectively, as candidates and regulatory elements. Thus, the rationale for selecting plant miR8126-3p and miR8126-5p for in vitro experiments was that (1) *QKI* and *MAPKAPK2* were predicted as putative targets of miR8126-3p and miR8126-5p, respectively, by all the prediction algorithms used, which could increase the probability of a real miRNA–gene interaction, and (2) *QKI* and *MAPKAPK2* have been previously associated with lipid metabolism [33,34,35,36,37,38]. The predicted miRNA–target gene interactions, potential interest, and biological impact on (lipid) metabolism were evaluated in human HepG2 hepatocytes in vitro.

### 2.2. Plant miR8126-3p and miR8126-5p Were Detected in HepG2 Cells after Transfection

Transfection efficiency was assessed 6 h post-transfection of 50 nM of plant miR8126-3p and miR8126-5p and a scramble sequence as a negative control (Supplementary Figure S2) in HepG2 cells. Undetectable levels of miR8126-3p and miR8126-5p were found in negative control cells, indicating that no human homologs of these plant miRNAs are encoded in the human hepatocyte HepG2 cell line. Remarkably, positive expression of plant miR8126-3p and miR8126-5p was achieved in HepG2 cells upon transfection with each of these mimics (Cq values between 17 and 30). This suggests that plant miRNAs are absent in basal conditions in human hepatocytes but are consistently present after mimic transfection, suggesting a high efficiency of our transfection procedure and experimental conditions.

### 2.3. Plant miR8126-3p and miR8126-5p Transfections Did Not Induce Cytotoxicity in HepG2 Cells

Cytotoxicity assays were conducted to avoid false positive results in subsequent analyses due to a potential cytotoxic effect of plant miRNA mimic treatments. First, cell viability was assessed with MTS (3-(4,5-dimethylthiazol-2-yl)-5-(3-carboxymethoxyphenyl)-2-(4-sulfophenyl)-2*H*-tetrazolium) in HepG2 cells transfected for 48 h with 50 nM of plant miR8126-3p and miR8126-5p mimics and a scramble sequence as a negative control (Supplementary Figure S3). This evaluation showed that plant miRNA mimics did not affect cell viability in HepG2 cells when compared with control cells. Cell viability was also evaluated in HepG2 cells treated with free fatty acids (FFAs) for 3 h after 48 h of transfection (Supplementary Figure S3). Cell viability was not modified in HepG2 cells transfected with either plant miR8126-3p or miR8126-5p and treated with FFAs, in comparison with their control (cells transfected with a scramble sequence and FFA-treated). However, a slight decrease (−7.70% ± 2.25; *p* < 0.05) in cell viability was reported when cell viability of the untreated control (cells transfected with a scramble sequence and not treated with FFAs) was compared with the FFA-treated control (cells transfected with a scramble sequence and treated with FFAs). Therefore, these plant miRNA mimic transfection conditions were applied in further studies to evaluate the impact of plant miRNAs in untreated or FFA-treated HepG2 cells due to their non-cytotoxic effect when compared with their control counterparts.

### 2.4. Plant miR8126-3p and miR8126-5p Modulated the Expression of Putative Target Genes and Metabolism-Associated Genes in HepG2 Cells

The effect of plant miRNAs on the expression of putative target genes and hepatocyte key metabolic genes was investigated after 48 h of mimic transfections. Specifically, we investigated the relative expression of *QKI* and *MAPKAPK2* (putative target genes of miR8126-3p and miR8126-5p, respectively) and other genes involved in glucose and lipid metabolism, including transcription factors and functional protein-coding genes. Moreover, gene expression was evaluated in HepG2 cells transfected for 48 h and then treated with FFAs for 3 h, in order to determine the impact of plant miRNA in a cell model that mimicked hepatic steatosis.

**Figure 1:**
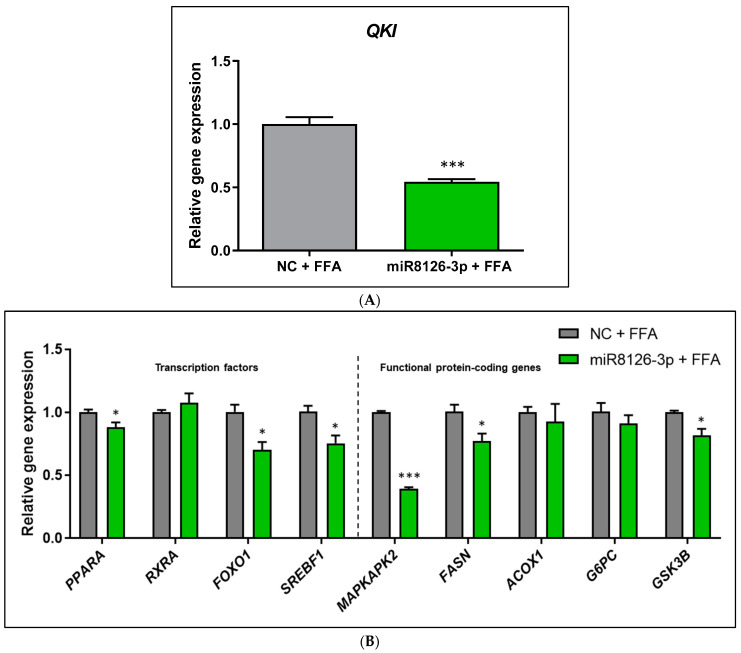
Gene expression analyses of HepG2 cells transfected with plant miR8126-3p mimic and treated with free fatty acids to mimic hepatic steatosis in vitro. (**A**) mRNA levels of the putative target gene *QKI*. (**B**) mRNA levels of transcription factors (*PPARA*, *RXRA*, *FOXO1*, *SREBF1*) and functional protein-coding genes (*MAPKAPK2, FASN*, *ACOX1*, *G6PC*, and *GSK3B*) involved in glucose and lipid metabolism. HepG2 cells were transfected with 50 nM of the mirVana mimic miR8126-3p (5′-UUCAGUAUUUUGACUCAGAA-3′) and a scramble sequence as a control (Negative Control #1). Forty-eight hours after transfection, cells were treated for 3 h with a mixture of free fatty acids (0.5 mM, proportion oleic:palmitic acids 2:1). mRNA expression levels were quantified using qPCR. Cq values were normalized to the housekeeping gene *TBP* (TATA-box binding protein) and expressed as the gene expression levels relative to the negative control cells treated with free fatty acids, calculated using the 2^−ΔΔCt^ method. Results are presented as the relative gene expression mean ± standard error of the mean (SEM) (n = 4–5). * *p* < 0.05, *** *p* < 0.001. Significance was determined by applying Student *t*-test (two-tailed). Abbreviations: NC (negative control), FFAs (free fatty acids), *QKI* (Quaking Homolog, KH Domain RNA Binding), *MAPKAPK2* (MAPK Activated Protein Kinase 2), *PPARA* (Peroxisome Proliferator Activated Receptor Alpha), *RXRA* (Retinoid X Receptor Alpha), *FOXO1* (Forkhead Box O1), *SREBF1* (Sterol Regulatory Element Binding Transcription Factor 1), *FASN* (Fatty Acid Synthase), *ACOX1* (Acyl-CoA Oxidase 1), *G6PC* (Glucose-6-Phosphatase Catalytic Subunit 1), *GSK3B* (Glycogen Synthase Kinase 3 Beta).

On one hand, the miR8126-3p mimic downregulated the mRNA expression of the putative target gene *QKI* (−45.85% ± 5.22; *p* < 0.001) in FFA-treated HepG2 cells (Figure 1A), which was accompanied by a similar decrease in the mRNA levels of *PPARA* (−12.05% ± 4.78; *p* < 0.05), *FOXO1* (−30.50% ± 8.86; *p* < 0.05), *SREBF1* (−25.10% ± 8.26; *p* < 0.05), *MAPKAPK2* (−61.20% ± 2.53; *p* < 0.001), *FASN* (−23.50% ± 8.63; *p* < 0.05), and *GSK3B* (−18.20% ± 5.95; *p* < 0.05). *RXRA*, *ACOX1*, and *G6PC* mRNA levels were not modified in comparison to the negative control cells (Figure 1B). Notably, a marked downregulation of predicted miR8126-3p target *QKI* mRNA (−36.29% ± 4.16; *p* < 0.001) was reported in basal (no FFA treatment) HepG2 cells as compared to control cells (Supplementary Figure S4A). Non-FFA-treated HepG2 cells also exhibited a significant decrease in mRNA expression of *PPARA* (−27.00% ± 4.47; *p* < 0.05), *FOXO1* (−39.57% ± 3.86; *p* < 0.001), *SREBF1* (−27.00% ± 6.44; *p* < 0.01), *MAPKAPK2* (−57.20% ± 4.78; *p* < 0.001), *FASN* (−27.57% ± 7.97; *p* < 0.01), *ACOX1* (−16.00% ± 2.24; *p* < 0.05), and *GSK3B* (−31.50% ± 3.64; *p* < 0.05), while no variation in mRNA expression of *RXRA* and *G6PC* was detected (Supplementary Figure S4B).

**Figure 2:**
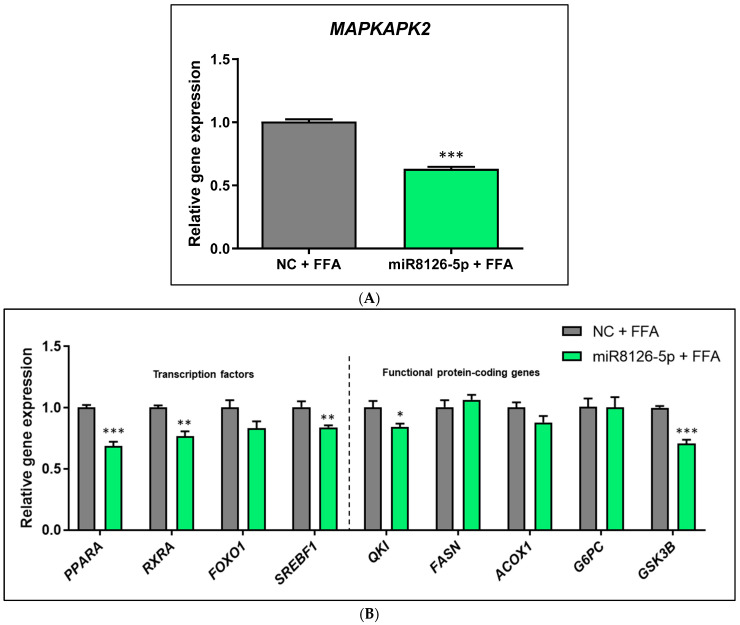
Gene expression analyses of HepG2 cells transfected with plant miR8126-5p mimic and treated with free fatty acids to mimic hepatic steatosis in vitro. (**A**) mRNA levels of the putative target gene *MAPKAPK2*. (**B**) mRNA levels of transcription factors (*PPARA*, *RXRA*, *FOXO1*, *SREBF1*) and functional protein-coding genes (*QKI*, *FASN*, *ACOX1*, *G6PC*, and *GSK3B*) involved in glucose and lipid metabolism. HepG2 cells were transfected with 50 nM of the mirVana mimic miR8126-5p (5′-UCUGAGUCAGAUUACUGAAUA-3′) and a scramble sequence as a control (Negative Control #1). Forty-eight hours after transfection, cells were treated for 3 h with a mixture of free fatty acids (0.5 mM, proportion oleic:palmitic acids 2:1). mRNA expression levels were quantified using qPCR. Cq values were normalized to the housekeeping gene *TBP* (TATA-box binding protein) and expressed as the gene expression levels relative to the negative control cells treated with free fatty acids, calculated using the 2^−ΔΔCt^ method. Results are presented as the relative gene expression mean ± standard error of the mean (SEM) (n = 4–5). * *p* < 0.05, ** *p* < 0.01, *** *p* < 0.001. Significance was determined by applying Student *t*-test (two-tailed). Abbreviations: NC (negative control), FFAs (free fatty acids), *QKI* (Quaking Homolog, KH Domain RNA Binding), *MAPKAPK2* (MAPK Activated Protein Kinase 2), *PPARA* (Peroxisome Proliferator Activated Receptor Alpha), *RXRA* (Retinoid X Receptor Alpha), *FOXO1* (Forkhead Box O1), *SREBF1* (Sterol Regulatory Element Binding Transcription Factor 1), *FASN* (Fatty Acid Synthase), *ACOX 1* (Acyl-CoA Oxidase 1), *G6PC* (Glucose-6-Phosphatase Catalytic Subunit 1), *GSK3B* (Glycogen Synthase Kinase 3 Beta).

On the other hand, the miR8126-5p mimic inhibited the expression of the putative target gene *MAPKAPK2* (−37.60% ± 2.51; *p* < 0.001) at the mRNA level in the in vitro model that mimicked hepatic steatosis (Figure 2A). The same cells exhibited a significant decrease in *PPARA* (−31.58% ± 4.80; *p* < 0.001), *RXRA* (−23.42% ± 5.21; *p* < 0.01), *SREBF1* (−16.50% ± 4.36; *p* < 0.01), *QKI* (−15.92% ± 5.39; *p* < 0.05), and *GSK3B* (−29.33% ± 4.25; *p* < 0.001) mRNA expression, while no significant changes were detected for *FOXO1*, *FASN*, *ACOX1*, and *G6PC* transcript levels (Figure 2B). miR8126-5p also modulated gene expression in basal (non-FFA-treated) HepG2 cells, where a significant inhibition of *MAPKAPK2* (−28.05% ± 4.54; *p* < 0.001) mRNA levels was induced (Supplementary Figure S5A). miR8126-5p also reduced *RXRA* (−16.43% ± 4.95; *p* < 0.01) and *SREBF1* (−31.00% ± 5.49; *p* < 0.001) mRNA levels (Supplementary Figure S5B). No significant changes were detected for *PPARA*, *FOXO1*, *QKI*, *FASN*, *ACOX1*, and *GSK3B* mRNA expression. Nevertheless, a trend towards the downregulation of *GSK3B* (−31.83% ± 12.68, *p* = 0.0870) was observed (Supplementary Figure S5B).

### 2.5. Plant miR8126-3p and miR8126-5p Decreased Lipid Accumulation in a Human Hepatocyte Cell Model That Mimicked Steatosis

To determine whether the modulation of genes involved in glucose and lipid metabolism induced by plant miRNAs could promote functional changes related to the prevention of lipid accumulation, we measured the intracellular triglyceride content in miRNA-mimic-transfected HepG2 cells.

**Figure 3:**
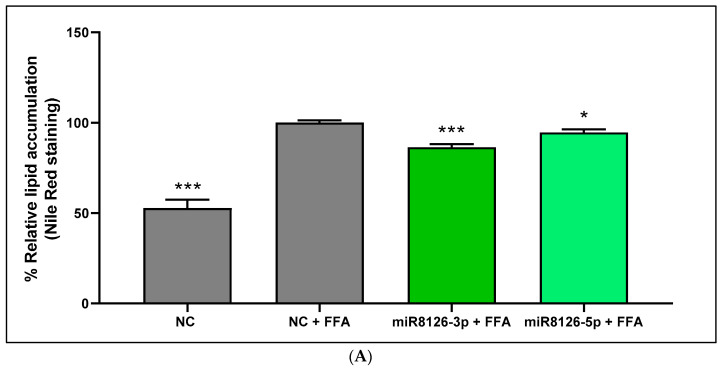

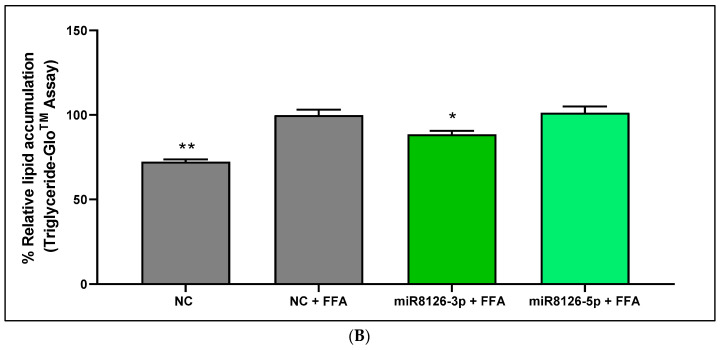
Quantification of intracellular triglycerides in HepG2 cells transfected with plant miR8126-3p and miR8126-5p mimics. (**A**) Quantification of intracellular triglycerides using Nile Red staining. (**B**) Quantification of intracellular triglycerides using Triglyceride-Glo^TM^ Assay. HepG2 were transfected for 48 h with 50 nM of mirVana mimics miR8126-3p (5′-UUCAGUAUUUUGACUCAGAA-3′) and miR8126-5p (5′-UCUGAGUCAGAUUACUGAAUA-3′) and a scramble sequence as a control (Negative Control #1). Forty-eight hours after transfections, cells were treated with 0.5 mM of free fatty acids (proportion oleic:palmitic acids 2:1) for 3 h. Cells transfected with a scramble sequence for 48 h, untreated with free fatty acids, were used as a positive control. Results are presented as the % lipid accumulation, which refers to the mean percentage ± standard error of the mean (SEM) of the triglyceride content relative to the negative control treated with free fatty acids (Nile Red staining: n = 10–11; Triglyceride-Glo^TM^ Assay: n = 3). Significance, determined by applying Student *t*-test (two-tailed), refers to the comparison of each plant miRNA mimic and untreated control cells with respect to the control treated with free fatty acids. * *p* < 0.05, ** *p* < 0.01, *** *p* < 0.001. Abbreviations: NC (negative control), FFAs (free fatty acids).

To validate the establishment of the HepG2 model that mimicked hepatic steatosis, comparisons were made between the negative control cells untreated and treated with FFAs. We observed that a 3 h treatment with FFAs induced a significant increase in lipid content accumulation in control (basal) hepatocytes (47.23% ± 4.65; *p* < 0.001) as quantified using Nile Red staining (Figure 3A). The plant miR8126-3p mimic diminished FFA-induced lipid accumulation (−13.59% ± 2.19; *p* < 0.001) as compared with the FFA-treated negative control (Figure 3A). Similarly, the miR8126-5p mimic also slightly decreased (−5.36% ± 2.09; *p* < 0.05) the lipid content after FFA treatment in HepG2 cells as compared to the FFA-treated negative control (Figure 3A).

Likewise, similar results were obtained with the Triglyceride-Glo^TM^ Assay (Figure 3B): FFA treatment in negative control cells increased lipid deposition (27.63% ± 3.48; *p* < 0.01) in comparison with untreated negative control (Figure 3B). The decrease in the lipid content observed using Nile Red staining in HepG2 cells transfected with miR8126-3p and miR8126-5p was only confirmed with the Triglyceride-Glo^TM^ Assay for miR8126-3p (−11.44% ± 3.77; *p* < 0.05), while no differences were observed for the miR8126-5p treatment compared to the FFA-treated negative control (Figure 3B).

## 3. Discussion

Plant food has been extensively associated with benefits in NAFLD management, and plant miRNAs have recently emerged as bioactive components with cross-kingdom regulatory functions [15,17]. Based on this evidence, in the present article, we evaluated the potential impact of plant miRNAs on the expression of glucose and lipid metabolic genes and lipid accumulation, a crucial triggering factor of hepatic steatosis.

Our in silico analyses, conducted to identify human putative target genes of plant miRNAs, revealed that plant miR8126 could potentially interact with genes related to energy metabolism, *QKI* [33,34,35] and *MAPKAPK2* [36,37,38]. However, dissimilarities in the sequences of the miR8126-3p and miR8126-5p isoforms (only sharing a 31% similarity) translated into different putative mRNA targets: miR8126-3p could potentially target *QKI*, which encodes for an RNA-binding protein involved in RNA processing, including the regulation of mRNA alternative splicing, stability, and translation [39]. On the other hand, a predicted target of plant miR8126-5p was *MAPKAPK2* (or *MK2*), which encodes for a protein kinase that acts through the p38 mitogen-activated protein kinase (p38 MAPK) pathway to regulate a plethora of cellular processes [40,41]. Our hypothesis is that miR8126-3p and miR8126-5p might have an impact on hepatocyte expression profile and lipid accumulation through the modulation of predicted target genes *QKI* and *MAPKAK2*, respectively. This hypothesis relies on several facts: (1) the miRNA mechanism of action after mRNA interaction is mainly inhibitory [18], and (2) *QKI* and *MAPKAPK2* inhibitions have been positively correlated with the prevention and/or counteraction of metabolic homeostasis disturbances including lipid alterations [34,37]. To validate this hypothesis, we used human hepatocytes (HepG2 cells) loaded with a mixture of FFAs (oleic and palmitic acids) as a model to mimic hepatic steatosis in vitro; this model was previously used in the study of plant bioactive compounds regulating lipid metabolism [42,43,44].

This study reports for the first time that plant miR8126-3p and miR8126-5p can inhibit the expression of their putative human target genes (*QKI* and *MAPKAPK2*, respectively) and have an impact on the mRNA levels of key genes involved in glucose and lipid metabolism, including transcription factors and functional protein-coding genes. Notably, both plant miRNAs also achieve the efficient modulation of predicted target and metabolic genes in basal (non-FFA-treated) HepG2 cells in a similar manner to that in FFA-treated cells. These findings collectively manifest that miR8126-3p and miR8126-5p could be plant-derived bioactive molecules that might modulate the metabolic gene expression profile in human hepatocytes in both baseline and FFA-overloading situations. Intriguingly, both miR8126 isoforms downregulated *QKI* and *MAPKAPK2* gene expression in the HepG2 model that mimicked hepatic steatosis, despite prediction algorithms reporting that miR8126-3p could target *QKI* and miR8126-5p could target *MAPKAPK2*, and not vice versa. Indeed, it could be speculated that both miR8126-3p and miR8126-5p might target *QKI* and *MAPKAPK2* since their sequences share a certain degree of similarity. This could be explained by the fact that bioinformatics predictions are based on global approximations and small mismatches could go unnoticed. However, the eventual/putative cross-regulation between plant miRNA–target interactions remains to be established in future studies.

Additionally, our aim was to have an overview of the eventual gene expression changes promoted by plant miR8126-3p and miR8126-5p. In FFA-treated HepG2 cells, both miRNAs promoted a marked downregulation of the mRNA levels of *SREBF1*, a transcription factor that regulates de novo lipogenesis in hepatocytes [45]. In concordance, a decrease in *FASN* gene expression was promoted by miR8126-3p. It has been described that the Retinoid X receptor (encoded by the *RXRA* gene) can form heterodimers with PPARα and stimulate the expression of *SREBF1* in hepatocytes [46,47,48]. In this study, both plant miR8126 isoforms downregulated *PPARA* gene expression, and miR8126-5p also decreased *RXRA* mRNA levels, which could explain the inhibition of the master regulator of lipogenesis *SREBF1*. As far as we know, a direct influence of the predicted targets of both isoforms of miR8126 miRNAs, *QKI* and *MAPKAPK2,* on the PPARA-RXRA pathway in hepatocytes has not been unveiled yet. Nonetheless, recent evidence showed that changes in *QKI* expression had an impact on LXR/RXR and PPAR activation and signaling in other cell types, in agreement with our results [35,49]. Further investigation will be needed to elucidate if *QKI* and *MAPKAPK2* inhibition promoted by plant miRNAs in hepatocytes could have a direct impact on *PPARA*/*RXRA* gene expression, which in turn might explain, at least in part, the downregulation of *SREBF1*. However, we do not exclude the possibility that plant miR8126-3p and miR8126-5p could act through other additional/complementary pathways to PPARA-RXRA, which could also contribute to the decrease observed in *SREBF1* mRNA levels [50,51]

We demonstrated that metabolic gene expression changes promoted by plant miR8126-3p and miR8126-5p translated into a reduction in lipid accumulation in the HepG2 model that mimicked steatosis. Transfections with plant miRNAs were performed prior to the FFA treatment, which suggests a potential preventive role of plant miRNAs attenuating triglyceride accumulation, as has been already observed for other plant bioactive compounds [52,53]. Two independent triglyceride quantification methods were used, Nile Red staining and the Triglyceride-Glo^TM^ Assay. However, the Nile Red staining method (performing several independent experiments with a large number of replicates) was considered more reliable since it is commonly used to efficiently quantify neutral lipids and evaluate the accumulation of triglycerides [54]. FFA treatment slightly decreased cellular viability (8%), although the concentration of oleic and palmitic acids used in our study is commonly used, even for longer periods of time, and was within the range of the physiological concentration of fatty acids in humans with non-alcoholic steatohepatitis [55,56,57]. Our Nile Red staining results demonstrated that miR8126-3p and miR8126-5p attenuated FFA-induced lipid deposition and evidenced slight differences between both miRNAs. The strongest effect was observed with miR8126-3p as compared with miR8126-5p treatments (14% vs. 5% of lipid content decrease), which may correlate with (and be eventually secondary to) the more prominent inhibition of *SREBF1* promoted by miR8126-3p, which also inhibited *FASN*. Despite small differences in the sensitivity of assays and techniques, the Triglyceride-Glo^TM^ Assay technique also corroborated the inhibitory capacity of miR8126-3p. Importantly, *QKI* inhibition has been related to decreased lipid accumulation in adipocytes, in agreement with our results [34]. Our results might suggest that plant miRNAs could attenuate fat deposition by inhibiting lipid-metabolism specific transcription factors (*SREBF1*), as previously suggested for other plant bioactive compounds (such as resveratrol), and thus could be applied for the treatment of NAFLD [58]. However, we did not assess the temporal dynamics of how plant miRNA targets interact with candidate genes and eventually, transcription factors and/or functional genes. Globally, our results suggest that plant miRNAs modulate gene expression and have an impact on metabolic cell function, translating into decreased lipid accumulation in hepatocytes in vitro.

Compelling evidence has already demonstrated the outstanding cross-kingdom effects of plant miRNAs on the glucose and lipid metabolism of mammalian cells, including the regulation of glucose uptake, fat synthesis, and cholesterol levels [26,59,60,61]. Our results are supported by previously published studies where, for instance, small RNAs from *Olea europaea* and *Moringa oleifera* decreased intracellular lipid accumulation in HepG2 hepatocytes [62,63]. We have unveiled for the first time the effect of plant miR8126-3p and miR8126-5p on the alleviation of triglyceride accumulation induced by FFAs in HepG2 cells, through the regulation of the predicted targets *QKI* and *MAPKAPK2*; these targets are widely associated with lipid metabolism, and their inhibition is associated with the counteraction of metabolic diseases [34,35,38]. Altogether, our findings suggest a promising therapeutic role of plant miRNAs in the counteraction of lipid metabolism dysregulation.

This study is not free of limitations: (1) Further in-depth studies will be required to determine the metabolic pathways modulated by miR8126-3p and miR8126-5p. (2) It will be necessary to evaluate if these plant miRNAs could modulate the expression of the predicted targets and hepatocyte metabolic genes beyond the mRNA level. (3) Transient transfection was performed to evaluate the impact of miRNA mimics in vitro since it is an efficient and commonly used method to study the mechanism of action of microRNAs, including plant microRNAs [64,65]. Nevertheless, more physiological conditions could be considered in future studies, such as the delivery of plant miRNAs in extracellular vesicles [66]. (4) Notably, the results obtained should be complemented in more cell lines, such as HuH 7, HepaRG, hepatocyte-like cells (HLCs), and/or primary human hepatocytes, since only HepG2 cells were used in this study [67]. (5) Importantly, *Prunus persica* was selected as a reference genome: choosing a well-identified genome (a reference miRNA set) to identify conserved miRNAs in other plant species is a commonly used strategy since miRNAs are widely conserved across the plant kingdom [68,69]. In this sense, it would be relevant to determine if plant miR8126-3p and miR8126-5p are detected in other edible plant-based products apart from peaches. (6) Additionally, the bioavailability of miR8126-3p and miR8126-5p in the gastrointestinal tract, circulatory system, and liver should be evaluated in animals and/or humans, along with their potential ability to ameliorate liver steatosis in vivo. Nevertheless, the results of our study pave the way for further research on new therapeutic approaches based on the potential use of plant miR8126 miRNAs to maintain hepatic metabolic homeostasis and prevent lipid dysregulation.

## 4. Materials and Methods

### 4.1. Bioinformatic Approach to Identify Putative Human Targets for Plant miRNAs

The microRNA database miRBase (https://www.mirbase.org; accessed on 12 April 2022) was employed to search for mature plant miRNA sequences: *Prunus persica* was used as a reference species, and its mature miRNA sequences registered in miRBase (last updated on 11 March 2018) were downloaded in FASTA format. The web servers TAPIR (http://bioinformatics.psb.ugent.be/webtools/tapir; accessed on 13 April 2022)) [31] and psRNATarget scoring schemas V1 and V2 (https://www.zhaolab.org/psRNATarget; accessed on 12 April 2022) [30] were employed to predict human target genes of plant miRNAs: The cDNA library “Homo sapiens (human), transcript, Human genomic sequencing project” (available on psRNATarget server) was chosen as target genome for both psRNATarget and TAPIR analyses. Bioinformatic predictions were carried out by applying the default parameters, with the exception that the number of top target genes was set to one million in psRNATarget scoring schemas V1 and V2. The parameters reported when applying TAPIR include the score (penalty for mismatches, G-U pairs, and gaps) and the minimum free energy (MFE) ratio, which compares the free energy of the miRNA:mRNA duplex to that of the same duplex with perfect matches [31]. The psRNATarget server provides the target accession number of the putative target genes, the expectation value (penalty for the mismatches in the alignment between the miRNA and the putative target sequence), the mRNA target aligned fragment, and the inhibitory effect (translation inhibition or cleavage) [30,70]. The Scoring Schema V1 also considers the maximum energy to unpair the target site (UPE), which represents the energy required to open the secondary structure that could be around the target site of the mRNA.

The web-based tool GeneCodis (https://GeneCodis.genyo.es; accessed on 15 April 2022) [71] was used to perform Gene Ontology (GO) enrichment analysis of predicted human target genes.

Candidate plant miRNAs and putative human target genes were selected according to the following criteria: (1) Only plant miRNAs with putative targets that appeared “present” in the three prediction algorithms (TAPIR and psRNATarget scoring schemas V1 and V2) were selected for literature search. (2) A bibliography search in the PubMed database (https://pubmed.ncbi.nlm.nih.gov; accessed on 15-28 April 2022) was conducted to select those miRNAs whose putative targets had been previously associated with lipid and/or liver metabolism and/or NAFLD.

### 4.2. Cell Culture and Plant miRNA Mimic Transfection

The human hepatoma HepG2 cell line (American Type Culture Collection, ATCC^®^ HB-8065™; Manassas, VA, USA) was grown in Dulbecco’s Modified Eagle Medium GlutaMAX-I (DMEM) (Gibco, Thermo Fisher Scientific Inc., Waltham, MA, USA) supplemented with 10% fetal bovine serum (FBS; Gibco, Thermo Fisher Scientific Inc.) and 1% penicillin–streptomycin solution (Gibco, Thermo Fisher Scientific Inc.) in an incubator at 37 °C and 5% CO_2_ until experiments were performed.

HepG2 cells were reverse-transfected with Lipofectamine RNAiMAX Reagent (Thermo Fisher Scientific Inc.) and 50 nM of the following mirVana™ miRNA mimics (Thermo Fisher Scientific Inc.): a scramble sequence as a negative control (mirVana™ miRNA Mimic, Negative Control #1), miR8126-3p (5′-UUCAGUAUUUUGACUCAGAA-3′; assay ID MC29889), and miR8126-5p (5′-UCUGAGUCAGAUUACUGAAUA-3′; assay ID MC27145). The miRNA mimics were diluted in Opti-MEM I Reduced Serum Medium (Gibco, Thermo Fisher Scientific Inc.) and added to well plates following manufacturer instructions. Next, lipofectamine was added to well plates containing the diluted miRNA mimics and incubated for 15 min at room temperature to form miRNA mimic–lipofectamine complexes inside the wells. Immediately, HepG2 cells diluted in DMEM supplemented with 10% FBS without antibiotics were plated. Cells were incubated for 6 h to evaluate the transfection efficiency (miRNA expression assays) and/or 48 h for the evaluation of mRNA expression, the evaluation of cytotoxicity, Red Nile staining, and triglyceride quantification assays. The volumes of the reagents and the number of cells used for HepG2 transfections are detailed in Supplementary Table S3.

To induce lipid accumulation and mimic steatosis [42,43], HepG2 cells were treated with a mixture of free fatty acids (FFAs) 48 h after miRNA mimic transfections: oleic and palmitic acids were mixed in DMEM GlutaMAX-I supplemented with 10% FBS, without antibiotics (proportion oleic:palmitic acids 2:1), and incubated with HepG2 cells for 3 h (48 h post-transfection) in a final concentration of 0.5 mM, which is in the range of the physiological concentration of fatty acids used to mimic hepatic steatosis [57].

### 4.3. RNA Isolation and Gene Expression Analyses

HepG2 cells were frozen on dry ice and stored at −80 °C until RNA extraction. Cells used for miRNA expression analyses were washed previously with phosphate-buffered saline (PBS) to completely remove potential unabsorbed miRNA mimics. Total RNA from HepG2 cells was isolated using TRIzol Reagent (Thermo Fisher Scientific Inc.) according to the manufacturer’s protocol. RNA concentration and purity (260/280 ratio) were determined using a NanoDrop ND-1000 spectrophotometer (Thermo Fisher Scientific Inc.).

Transfection efficiency was evaluated after 6 h of HepG2 incubation with miRNA mimic–lipofectamine complexes, by analyzing the expression of miR8126-3p and miR8126-5p. Retro-transcription of total RNA (4 µL) into cDNA was performed with miRCURY LNA RT Kit (Qiagen, Hilden, Germany) in a final volume reaction of 10 µL. Reactions were conducted in a MyCycler Thermal Cycler (Bio-Rad, Hercules, CA, USA) at 42 °C for 60 min and 95 °C for 5 min. Quantitative-real time PCR (qPCR) was carried out with cDNA diluted 1/10, miRCURY LNA SYBR Green PCR Kit (Qiagen), and the following miRCURY LNA miRNA PCR Assays (Qiagen): miR8126-3p (5′-UUCAGUAUUUUGACUCAGAA-3′; GeneGlobe Id: YP02100033) and miR8126-5p (5′-UCUGAGUCAGAUUACUGAAUA-3′; GeneGlobe Id: YP02115447). QPCR reactions were performed using a CFX384 Touch Real-Time PCR Detection System (Bio-Rad), applying the following conditions: 95 °C for 2 min, 40 cycles at 95 °C for 10 s, and 56 °C for 1 min.

mRNA expression was evaluated in HepG2 cells transfected with miRNA mimics for 48 h, non-treated and treated with FFAs (for 3 h). RNA (1 µg) was treated with DNA-free™ DNA Removal Kit (Invitrogen. Thermo Fisher Scientific Inc.), according to the manufacturer’s instructions. For reverse transcription, 5 ng/µL of Random Primers (Invitrogen. Thermo Fisher Scientific Inc.) and 250 µM of dNTP Mix (Bioline, Luckenwalde, Germany) were added into DNA-free RNA and incubated for 5 min at 65 °C. Then, 2 units/µL of Recombinant RNAsin Ribonuclease Inhibitor (Promega, Madison, WI, USA), 10 mM of dithiothreitol (DTT; Invitrogen. Thermo Fisher Scientific Inc.), and 1X First-Strand buffer (Invitrogen. Thermo Fisher Scientific Inc.) were added to each tube and incubated for 2 min at 37 °C. Then, 10 units/µL of M-MLV Reverse Transcriptase (Invitrogen. Thermo Fisher Scientific Inc.) was added to each sample and incubated at 25 °C for 10 min, 37 °C for 50 min, and 70 °C for 15 min. All the reactions were conducted in a GeneAmp PCR System 2700 (Applied Biosystems. Thermo Fisher Scientific Inc.). QPCR was performed with cDNA diluted 1/1.5 using iTaq™ Universal Probes Supermix (Bio-Rad) and specific predesigned assays from Integrated DNA Technologies (IDT; Coralville, IA, USA) and Taqman (Thermo Fisher Scientific Inc.), which are described in Table 3. QPCR reactions were performed in a CFX384 Touch Real-Time PCR Detection System (Bio-Rad), applying the following cycling conditions: 50 °C for 2 min, 95 °C for 10 min, 40 cycles at 95 °C for 15 s, and 60 °C for 1 min. The gene TATA-box binding protein (*TBP*) was used as a housekeeping control to normalize gene expression levels (∆Cq). Comparisons between gene expression levels after plant miRNA mimic transfections vs. scramble-sequence-transfected cells (negative control) were established using the 2^−∆∆Cq^ method, determining relative gene expression [72].

### 4.4. Cytotoxicity Assays

HepG2 cell viability was evaluated using an MTS (3-(4,5-dimethylthiazol-2-yl)-5-(3-carboxymethoxyphenyl)-2-(4-sulfophenyl)-2*H*-tetrazolium) colorimetric assay. Cytotoxicity assays were performed in HepG2 cells transfected for 48 h, non-treated and treated with FFAs for 3 h. MTS solution (CellTiter 96^®^ Aqueous One Solution Cell Proliferation Assay. Promega) was added to the cells (20 µL of MTS per 100 µL of culture medium) and incubated for 2.5 h at 37 °C. An Agilent BioTek 800 TS microplate reader (Agilent Technologies, Santa Clara, CA, USA) was used to measure the absorbance at 492 nm. Cell viability was expressed as the % relative viability, calculated as follows: mean of the absorbance of the experimental group × 100/mean of the absorbance of negative control. Comparisons between scramble-sequence-transfected cells (negative control) and experimental groups were expressed as the % relative variability.

### 4.5. Intracellular Triglyceride Quantification

Nile Red staining was performed to quantify the intracellular triglyceride content of HepG2 cells transfected (48 h) with miRNA mimics, treated and non-treated with FFAs (3 h). Cells were washed twice with PBS and fixed with 3.7% formaldehyde for 40 min. Afterward, cells were washed with 60% isopropanol for 3 min, dried for 30 min, and incubated with 200 µL of Nile Red Solution (N3013. Sigma-Aldrich, San Luis, MO, USA) diluted in PBS at 5 µg/mL, for 25 min, with mild shaking in the dark. Cells were washed three times with PBS, and fluorescence was quantified in a Fluoroskan Ascent FL (Thermo Fisher Scientific Inc.) using 544 nm excitation and 635 nm emission filters. All the steps were performed at room temperature. Of note, Nile Red stock solution (1 mg/mL) was prepared in acetone, shaken mildly overnight in the dark, and filtered with 0.22 µm pore filters, at least 48 h before use. The degree of lipid accumulation was calculated as the fluorescence emission of each experimental group relative to the negative control cells (% relative lipid accumulation).

To complement Nile Red staining assays, intracellular lipid content was also quantified using the Triglyceride-Glo^TM^ Assay (Promega), according to the manufacturer’s protocol. This assay quantifies triglyceride content by measuring glycerol levels. It was performed in HepG2 cells transfected for 48 h with miRNA mimics, treated and non-treated with FFAs for 3 h. Luminescence was quantified using white plates in a Fluoroskan Ascent FL (Thermo Fisher Scientific Inc.). Luminescence levels were expressed as the % relative lipid accumulation by establishing comparisons between experimental groups and negative control cells.

### 4.6. Statistical Analysis

Statistical analyses were conducted using GraphPad Prism 6.0 for Windows (GraphPad Software Inc., La Jolla, CA, USA), applying the Student *t*-test (two-tailed) to compare the experimental group (plant-miRNA-transfected cells) vs. negative control (cells transfected with a scramble sequence, non-treated or treated with FFAs). Differences were considered statistically significant at *p*-value < 0.05. The Student *t*-test was selected for statistical analyses because the objective of the study was to determine differences between miR8126-3p and miR8126-5p and the negative control, as independent studies, and not to establish a comparison between the three groups.

## 5. Conclusions

Plant-derived miR8126-3p and miR8126-5p could modulate the expression of human genes involved in hepatocyte metabolism, thereby improving the metabolic gene expression profile and alleviating lipid accumulation in basal and FFA-treated HepG2 cells. These new findings suggest that plant miRNAs could be promising bioactive molecules for preventing lipid homeostasis dysregulation in hepatocytes. Our results could pave the way for deeper investigations to evaluate the physiological impact of plant miR8126 miRNAs to further develop new therapeutic approaches for managing hepatic steatosis.

## Figures and Tables

**Table 1:** Putative human target genes of plant miR8126-3p predicted with the bioinformatic programs psRNATarget (scoring schemas V1 and V2) and TAPIR.

miR8126-3p Putative Human Target Genes				
**psRNATarget. Scoring Schema V1**				
**Target Accession**	**Expectation**	**UPE**	**mRNA Target Aligned Fragment (5′-3′)**	**Inhibitory Effect**
**NM_052929|*FHAD1***	2.5	20.471	63-[AUCUGUGUCAAAAUACUGAG]-82	Cleavage
NM_017864|*INTS8*	2.5	14.728	108-[UUUUCAGACAAAAUACUGAA]-127	Cleavage
**NM_001042625|*CAPSL***	3.0	15.911	110-[UUCUGUGUCAAAAUAUUGCA]-129	Cleavage
NM_007359|*CASC3*	3.0	20.567	276-[UUCUGAGUCUAGAUACAGAA]-295	Translation
NM_199355|*ADAMTS18*	3.0	12.676	1713-[UUUUGAGAUAAAAUGUUGAA]-1732	Cleavage
NM_001098634|*RBM47*	3.0	11.972	668-[UUCUGAGUCCAAACACUGGA]-687	Translation
**NM_006775|*QKI***	3.0	9.969	695-[AUCUGAGUUAAAAUUACUGAA]-715	Cleavage
**NM_206853|*QKI***	3.0	9.969	5434-[AUCUGAGUUAAAAUUACUGAA]-5454	Cleavage
**NM_206854|*QKI***	3.0	9.969	6695-[AUCUGAGUUAAAAUUACUGAA]-6715	Cleavage
**NM_206855|*QKI***	3.0	9.969	7660-[AUCUGAGUUAAAAUUACUGAA]-7680	Cleavage
NM_001104545|*TMEM255A*	3.0	14.245	2064-[UUCUGCUUUAAAGUACUGAA]-2083	Cleavage
**psRNATarget. Scoring Schema V2**				
**Target Accession**	**Expectation**	**UPE**	**mRNA Target Aligned Fragment (5′-3′)**	**Inhibitory Effect**
**NM_052929|*FHAD1***	1.5	N/A	63-[AUCUGUGUCAAAAUACUGAG]-82	Cleavage
NM_019644|*ANKRD7*	2.5	N/A	285-[AUUUGUGUCAAAAUGUUGAA]-304	Cleavage
NM_001099294|*KIAA1644*	2.5	N/A	418-[AUUUGUGUCAGAAUAUUGAA]-437	Cleavage
**NM_001042625|*CAPSL***	3.0	N/A	110-[UUCUGUGUCAAAAUAUUGCA]-129	Cleavage
NM_017864|*INTS8*	3.0	N/A	108-[UUUUCAGACAAAAUACUGAA]-127	Cleavage
NM_001270458|*MAOA*	3.0	N/A	697-[AACUGAGUUAGAAUGUUGAA]-716	Cleavage
NM_004690|*LATS1*	3.0	N/A	3251-[AUUUGGGUCAAAAUAUUGGU]-3270	Cleavage
NM_001104545|*TMEM255A*	3.0	N/A	2064-[UUCUGCUUUAAAGUACUGAA]-2083	Cleavage
NM_007359|*CASC3*	3.5	N/A	276-[UUCUGAGUCUAGAUACAGAA]-295	Translation
NM_199355|*ADAMTS18*	3.5	N/A	1713-[UUUUGAGAUAAAAUGUUGAA]-1732	Cleavage
NM_001098634|*RBM47*	3.5	N/A	668-[UUCUGAGUCCAAACACUGGA]-687	Translation
**NM_006775|*QKI***	3.5	N/A	695-[AUCUGAGUUAAAAUUACUGAA]-715	Cleavage
**NM_206853|*QKI***	3.5	N/A	5434-[AUCUGAGUUAAAAUUACUGAA]-5454	Cleavage
**NM_206854|*QKI***	3.5	N/A	6695-[AUCUGAGUUAAAAUUACUGAA]-6715	Cleavage
**NM_206855|*QKI***	3.5	N/A	7660-[AUCUGAGUUAAAAUUACUGAA]-7680	Cleavage
NM_000997|*RPL37*	3.5	N/A	752-[UUCUGUGUUAAAGCACUGAA]-771	Cleavage
NM_001040455|*SIDT2*	3.5	N/A	688-[UUCUGAUUCAAGAGGCUGAA]-707	Cleavage
NM_001039199|*TTPAL*	3.5	N/A	4980-[UUCUAAGUCAAAGUGGUGAA]-4999	Cleavage
NM_203395|*IYD*	3.5	N/A	738-[CAGUGAGUCAAGAUGCUGAG]-757	Cleavage
NM_001164694|*IYD*	3.5	N/A	838-[CAGUGAGUCAAGAUGCUGAG]-857	Cleavage
NM_001164695|*IYD*	3.5	N/A	925-[CAGUGAGUCAAGAUGCUGAG]-944	Cleavage
NM_012069|*ATP1B4*	4.0	N/A	3011-[UUUUGAGUUAAAAUAGUUAA]-3030	Cleavage
NM_134262|*RORA*	4.0	N/A	6935-[UUUUGAGUCAUAAUAUUUAA]-6954	Translation
NM_006311|*NCOR1*	4.5	N/A	1907-[UUCUGAGUCAGAAUACAGUU]-1926	Cleavage
**TAPIR**				
**Target Accession**	**Score**	**MFE Ratio**	**mRNA Target Aligned Fragment (5′-3′)**	
**NM_052929|*FHAD1***	2.5	0.8	63-[AUCUGUGUCAAAAUACUGAG]-82	
**NM_001042625|*CAPSL***	4.0	0.78	110-[UUCUGUGUCAAAAUAUUGCA]-129	
**NM_006775|*QKI***	4.0	0.75	695-[AUCUGAGUUAAAAUUACUGAA]-715	
**NM_206853|*QKI***	4.0	0.75	5434-[AUCUGAGUUAAAAUUACUGAA]-5454	
**NM_206854|*QKI***	4.0	0.75	6695-[AUCUGAGUUAAAAUUACUGAA]-6715	
**NM_206855|*QKI***	4.0	0.75	7660-[AUCUGAGUUAAAAUUACUGAA]-7680	

Mature sequence of plant miR8126-3p (5′-UUCAGUAUUUUGACUCAGAA-3′) was aligned with the cDNA library “*Homo sapiens* (human), transcript, Human genomic sequencing project” (from psRNATarget server). Predicted outputs highlighted **in bold** point out targets common to the three prediction algorithms. Abbreviations: UPE (unpaired energy), N/A (not applicable), MFE (minimum free energy).

**Table 2:** Putative human target genes of plant miR8126-5p predicted with the bioinformatic programs psRNATarget (scoring schemas V1 and V2) and TAPIR.

miR8126-5p Putative Human Target Genes				
**psRNATarget. Scoring Schema V1**				
**Target Accession**	**Expectation**	**UPE**	**mRNA Target Aligned Fragment (5′-3′)**	**Inhibitory Effect**
NM_001201365|*RNF14*	2.5	19.838	693-[AAUUUAGUGAUACUGGCUCAGA]-714	Translation
**NM_032960|*MAPKAPK2***	3.0	16.147	248-[UUAUCAGUAAUUUGACUUAGA]-268	Cleavage
**NM_004759|*MAPKAPK2***	3.0	16.147	875-[UUAUCAGUAAUUUGACUUAGA]-895	Cleavage
NM_057162|*KLHL4*	3.0	14.77	30-[CAUUUAUCAAUCUGACUCAGG]-50	Cleavage
NM_019117|*KLHL4*	3.0	17.45	2890-[CAUUUAUCAAUCUGACUCAGG]-2910	Cleavage
**psRNATarget. Scoring Schema V2**				
**Target Accession**	**Expectation**	**UPE**	**mRNA Target Aligned Fragment (5′-3′)**	**Inhibitory Effect**
**NM_032960|*MAPKAPK2***	2.0	N/A	248-[UUAUCAGUAAUUUGACUUAGA]-268	Cleavage
**NM_004759|*MAPKAPK2***	2.0	N/A	875-[UUAUCAGUAAUUUGACUUAGA]-895	Cleavage
NM_173654|*EOGT*	2.5	N/A	1618-[ACUUGAGUAGUCUGAUUUAGA]-1638	Cleavage
NM_057162|*KLHL4*	3.0	N/A	30-[CAUUUAUCAAUCUGACUCAGG]-50	Cleavage
NM_019117|*KLHL4*	3.0	N/A	2890-[CAUUUAUCAAUCUGACUCAGG]-2910	Cleavage
NM_006663|*PPP1R13L*	3.5	N/A	177-[AAUUUAGUAAUCUGCCUUAGC]-197	Cleavage
NM_001001343|*FNDC9*	3.5	N/A	708-[UAUUAAAUAAUCUGACUUAGC]-728	Cleavage
NM_020803|*KLHL8*	3.5	N/A	1383-[UGGUCAGUAAUCUGGUUCAUA]-1403	Cleavage
NM_002076|*GNS*	3.5	N/A	3204-[AUCUCAGUCAUUUGACUUAGA]-3224	Cleavage
NM_001144966|*NEDD4L*	4.0	N/A	4700-[UAUUGAGUAAUCUGGUUUCGA]-4720	Cleavage
NM_001146213|*TBC1D15*	4.0	N/A	1927-[GAUACAGUUAUUUGACUCAGU]-1947	Cleavage
NM_001201365|*RNF14*	4.5	N/A	693-[AAUUUAGUGAUACUGGCUCAGA]-714	Translation
NM_001168724|*TMEM135*	4.5	N/A	3417-[UAUUAAGUAGUUUGACACAGC]-3437	Cleavage
NM_207304|*MBNL2*	4.5	N/A	2298-[UAUUCAGAAGUCUGACUAUGA]-2318	Cleavage
NM_144778|*MBNL2*	4.5	N/A	2411-[UAUUCAGAAGUCUGACUAUGA]-2431	Cleavage
NM_001202485|*HSPE1-MOB4*	4.5	N/A	1820-[GAUUUGGUGAUCUGGCUGAGU]-1840	Cleavage
NM_001002909|*GPATCH8*	5.0	N/A	1921-[UGUUCAGUACUCAUCUGAUUCAGA]-1944	Cleavage
NM_030920|*ANP32E*	5.0	N/A	593-[UAUUCAGUAAUAUGGUUCAUG]-613	Translation
NM_001171909|*CXorf40A*	5.0	N/A	406-[UGUUCAGUGUUCUGACUCGCC]-426	Cleavage
NM_001041|*SI*	5.0	N/A	164-[UAUUAUAGUAAUGUGACUUGGA]-185	Translation
**TAPIR**				
**Target Accession**	**Score**	**MFE Ratio**	**mRNA Target Aligned Fragment (5′-3′)**	
**NM_032960|*MAPKAPK2***	4.0	0.76	248-[UUAUCAGUAAUUUGACUUAGA]-268	
**NM_004759|*MAPKAPK2***	4.0	0.76	875-[UUAUCAGUAAUUUGACUUAGA]-895	

Mature sequence of plant miR8126-5p (5′-UCUGAGUCAGAUUACUGAAUA-3′) was aligned with the cDNA library “*Homo sapiens* (human), transcript, Human genomic sequencing project” (available at psRNATarget server). Predicted outputs highlighted **in bold** represent targets common to the three prediction algorithms. Abbreviations: UPE (unpaired energy), N/A (not applicable), MFE (minimum free energy).

**Table 3:** Predesigned qPCR assays from Taqman (Thermo Fisher Scientific Inc.) (^1^) and Integrated DNA Technologies (^2^) used for gene expression analyses.

Gene Name	Assay ID	RefSeq
TBP	^1^Hs00427620_m1	NM_001172085.1NM_003194.4
QKI	^2^Hs.PT.58.2815647	NM_006775(1)
MAPKAPK2	^2^Hs.PT.58.2443418	NM_004759(2)
PPARA	^2^Hs.PT.58.45310483	NM_001001928(2)
RXRA	^2^Hs.PT.58.3784663	NM_002957(1)
FOXO1	^2^Hs.PT.58.40005627	NM_002015(1)
SREBF1	^2^Hs.PT.58.3359761	NM_001005291(2)
FASN	^1^Hs01005622_m1	NM_004104.4XM_011523538.2
ACOX1	^2^Hs.PT.56a.3058584	NM_001185039(3)
G6PC	^2^Hs.PT.58.5006581	NM_000151(2)
GSK3B	^2^Hs.PT.58.40111551	NM_001146156(2)

Abbreviations: RefSeq (Reference Sequence), *TBP* (TATA-box binding protein), *QKI* (Quaking Homolog, KH Domain RNA Binding), *MAPKAPK2* (MAPK Activated Protein Kinase 2), *PPARA* (Peroxisome Proliferator Activated Receptor Alpha), *RXRA* (Retinoid X Receptor Alpha), *FOXO1* (Forkhead Box O1)*, SREBF1* (Sterol Regulatory Element Binding Transcription Factor 1), *FASN* (Fatty Acid Synthase), *ACOX1* (Acyl-CoA Oxidase 1), *G6PC* (Glucose-6-Phosphatase Catalytic Subunit 1), *GSK3B* (Glycogen Synthase Kinase 3 Beta).

## Data Availability

The raw data supporting the conclusions of this article will be made available by the authors on request.

## Supplementary Materials

The following supporting information can be downloaded at: https://www.mdpi.com/article/10.3390/ijms25031721/s1.

## References

[1] Ipsen D.H., Lykkesfeldt J., Tveden-Nyborg P. (2018). Molecular mechanisms of hepatic lipid accumulation in non-alcoholic fatty liver disease. Cell. Mol. Life Sci..

[2] Younossi Z.M., Golabi P., Paik J.M., Henry A., Van Dongen C., Henry L. (2023). The global epidemiology of nonalcoholic fatty liver disease (NAFLD) and nonalcoholic steatohepatitis (NASH): A systematic review. Hepatology.

[3] Huh Y., Cho Y.J., Nam G.E. (2022). Recent Epidemiology and Risk Factors of Nonalcoholic Fatty Liver Disease. J. Obes. Metab. Syndr..

[4] Younossi Z., Anstee Q.M., Marietti M., Hardy T., Henry L., Eslam M., George J., Bugianesi E. (2018). Global burden of NAFLD and NASH: Trends, predictions, risk factors and prevention. Nat. Rev. Gastroenterol. Hepatol..

[5] Huang D.Q., El-Serag H.B., Loomba R. (2021). Global epidemiology of NAFLD-related HCC: Trends, predictions, risk factors and prevention. Nat. Rev. Gastroenterol. Hepatol..

[6] Liu Y., Zhong G.-C., Tan H.-Y., Hao F.-B., Hu J.-J. (2019). Nonalcoholic fatty liver disease and mortality from all causes, cardiovascular disease, and cancer: A meta-analysis. Sci. Rep..

[7] Lazarus J.V., Mark H.E., Anstee Q.M., Arab J.P., Batterham R.L., Castera L., Cortez-Pinto H., Crespo J., Cusi K., Dirac M.A. (2022). Advancing the global public health agenda for NAFLD: A consensus statement. Nat. Rev. Gastroenterol. Hepatol..

[8] Raza S., Rajak S., Upadhyay A., Tewari A., Anthony Sinha R. (2021). Current treatment paradigms and emerging therapies for NAFLD/NASH. Front. Biosci..

[9] Berná G., Romero-Gomez M. (2020). The role of nutrition in non-alcoholic fatty liver disease: Pathophysiology and management. Liver Int..

[10] Moore M.P., Cunningham R.P., Dashek R.J., Mucinski J.M., Rector R.S. (2020). A Fad too Far? Dietary Strategies for the Prevention and Treatment of NAFLD. Obesity.

[11] Yaskolka Meir A., Rinott E., Tsaban G., Zelicha H., Kaplan A., Rosen P., Shelef I., Youngster I., Shalev A., Blüher M. (2021). Effect of green-Mediterranean diet on intrahepatic fat: The DIRECT PLUS randomised controlled trial. Gut.

[12] Li X., Peng Z., Li M., Zeng X., Li H., Zhu Y., Chen H., Hu A., Zhao Q., Zhang Z. (2022). A Healthful Plant-Based Diet Is Associated with Lower Odds of Nonalcoholic Fatty Liver Disease. Nutrients.

[13] Bagherniya M., Nobili V., Blesso C.N., Sahebkar A. (2018). Medicinal plants and bioactive natural compounds in the treatment of non-alcoholic fatty liver disease: A clinical review. Pharmacol. Res..

[14] Sun Q., Xin X., An Z., Hu Y., Feng Q. (2022). Therapeutic Potential of Natural Plants Against Non-Alcoholic Fatty Liver Disease: Targeting the Interplay Between Gut Microbiota and Bile Acids. Front. Cell. Infect. Microbiol..

[15] Li H.-Y., Gan R.-Y., Shang A., Mao Q.-Q., Sun Q.-C., Wu D.-T., Geng F., He X.-Q., Li H.-B. (2021). Plant-Based Foods and Their Bioactive Compounds on Fatty Liver Disease: Effects, Mechanisms, and Clinical Application. Oxid. Med. Cell. Longev..

[16] Xie W., Weng A., Melzig M.F. (2016). MicroRNAs as New Bioactive Components in Medicinal Plants. Planta Med..

[17] Li D., Yang J., Yang Y., Liu J., Li H., Li R., Cao C., Shi L., Wu W., He K. (2021). A Timely Review of Cross-Kingdom Regulation of Plant-Derived MicroRNAs. Front. Genet..

[18] Bartel D.P. (2018). Metazoan MicroRNAs. Cell.

[19] Owusu Adjei M., Zhou X., Mao M., Rafique F., Ma J. (2021). MicroRNAs Roles in Plants Secondary Metabolism. Plant Signal. Behav..

[20] Dong Q., Hu B., Zhang C. (2022). microRNAs and Their Roles in Plant Development. Front. Plant Sci..

[21] Zhang F., Yang J., Zhang N., Wu J., Si H. (2022). Roles of microRNAs in abiotic stress response and characteristics regulation of plant. Front. Plant Sci..

[22] Zhao Q., Liu Y., Zhang N., Hu M., Zhang H., Joshi T., Xu D. (2018). Evidence for plant-derived xenomiRs based on a large-scale analysis of public small RNA sequencing data from human samples. PLoS ONE.

[23] Luo Y., Wang P., Wang X., Wang Y., Mu Z., Li Q., Fu Y., Xiao J., Li G., Ma Y. (2017). Detection of dietetically absorbed maize-derived microRNAs in pigs. Sci. Rep..

[24] Díez-Sainz E., Milagro F.I., Riezu-Boj J.I., Lorente-Cebrián S. (2021). Effects of gut microbiota–derived extracellular vesicles on obesity and diabetes and their potential modulation through diet. J. Physiol. Biochem..

[25] Dickinson B., Zhang Y., Petrick J.S., Heck G., Ivashuta S., Marshall W.S. (2013). Lack of detectable oral bioavailability of plant microRNAs after feeding in mice. Nat. Biotechnol..

[26] Zhang L., Hou D., Chen X., Li D., Zhu L., Zhang Y., Li J., Bian Z., Liang X., Cai X. (2012). Exogenous plant MIR168a specifically targets mammalian LDLRAP1: Evidence of cross-kingdom regulation by microRNA. Cell Res..

[27] Chen X., Liu L., Chu Q., Sun S., Wu Y., Tong Z., Fang W., Timko M.P., Fan L. (2021). Large-scale identification of extracellular plant miRNAs in mammals implicates their dietary intake. PLoS ONE.

[28] Zhang L., Chen T., Yin Y., Zhang C.-Y., Zhang Y.-L. (2019). Dietary microRNA-A Novel Functional Component of Food. Adv. Nutr..

[29] Saiyed A.N., Vasavada A.R., Johar S.R.K. (2022). Recent trends in miRNA therapeutics and the application of plant miRNA for prevention and treatment of human diseases. Futur. J. Pharm. Sci..

[30] Dai X., Zhao P.X. (2011). psRNATarget: A plant small RNA target analysis server. Nucleic Acids Res..

[31] Bonnet E., He Y., Billiau K., Van de Peer Y. (2010). TAPIR, a web server for the prediction of plant microRNA targets, including target mimics. Bioinformatics.

[32] Srivastava P.K., Moturu T.R., Pandey P., Baldwin I.T., Pandey S.P. (2014). A comparison of performance of plant miRNA target prediction tools and the characterization of features for genome-wide target prediction. BMC Genom..

[33] Zhou X., Shin S., He C., Zhang Q., Rasband M.N., Ren J., Dai C., Zorrilla-Veloz R.I., Shingu T., Yuan L. (2021). Qki regulates myelinogenesis through Srebp2-dependent cholesterol biosynthesis. Elife.

[34] Lu H., Ye Z., Zhai Y., Wang L., Liu Y., Wang J., Zhang W., Luo W., Lu Z., Chen J. (2020). QKI regulates adipose tissue metabolism by acting as a brake on thermogenesis and promoting obesity. EMBO Rep..

[35] Zhou X., He C., Ren J., Dai C., Stevens S.R., Wang Q., Zamler D., Shingu T., Yuan L., Chandregowda C.R. (2020). Mature myelin maintenance requires Qki to coactivate PPARβ-RXRα-mediated lipid metabolism. J. Clin. Investig..

[36] Ruiz M., Coderre L., Allen B.G., Des Rosiers C. (2018). Protecting the heart through MK2 modulation, toward a role in diabetic cardiomyopathy and lipid metabolism. Biochim. Biophys. Acta. Mol. Basis Dis..

[37] Ozcan L., Xu X., Deng S.-X., Ghorpade D.S., Thomas T., Cremers S., Hubbard B., Serrano-Wu M.H., Gaestel M., Landry D.W. (2015). Treatment of Obese Insulin-Resistant Mice with an Allosteric MAPKAPK2/3 Inhibitor Lowers Blood Glucose and Improves Insulin Sensitivity. Diabetes.

[38] Ruiz M., Coderre L., Lachance D., Houde V., Martel C., Thompson Legault J., Gillis M.-A., Bouchard B., Daneault C., Carpentier A.C. (2016). MK2 Deletion in Mice Prevents Diabetes-Induced Perturbations in Lipid Metabolism and Cardiac Dysfunction. Diabetes.

[39] Darbelli L., Richard S. (2016). Emerging functions of the Quaking RNA-binding proteins and link to human diseases. Wiley Interdiscip. Rev. RNA.

[40] Gaestel M. (2006). MAPKAP kinases—MKs—two’s company, three’s a crowd. Nat. Rev. Mol. Cell Biol..

[41] Trempolec N., Muñoz J.P., Slobodnyuk K., Marin S., Cascante M., Zorzano A., Nebreda A.R. (2017). Induction of oxidative metabolism by the p38α/MK2 pathway. Sci. Rep..

[42] Eynaudi A., Díaz-Castro F., Bórquez J.C., Bravo-Sagua R., Parra V., Troncoso R. (2021). Differential Effects of Oleic and Palmitic Acids on Lipid Droplet-Mitochondria Interaction in the Hepatic Cell Line HepG2. Front. Nutr..

[43] Gómez-Lechón M.J., Donato M.T., Martínez-Romero A., Jiménez N., Castell J.V., O’Connor J.-E. (2007). A human hepatocellular in vitro model to investigate steatosis. Chem. Biol. Interact..

[44] Kim S.H., Yun C., Kwon D., Lee Y.-H., Kwak J.-H., Jung Y.-S. (2023). Effect of Isoquercitrin on Free Fatty Acid-Induced Lipid Accumulation in HepG2 Cells. Molecules.

[45] Eberlé D., Hegarty B., Bossard P., Ferré P., Foufelle F. (2004). SREBP transcription factors: Master regulators of lipid homeostasis. Biochimie.

[46] Yoshikawa T., Shimano H., Amemiya-Kudo M., Yahagi N., Hasty A.H., Matsuzaka T., Okazaki H., Tamura Y., Iizuka Y., Ohashi K. (2001). Identification of liver X receptor-retinoid X receptor as an activator of the sterol regulatory element-binding protein 1c gene promoter. Mol. Cell. Biol..

[47] Gao W.-Y., Chen P.-Y., Hsu H.-J., Lin C.-Y., Wu M.-J., Yen J.-H. (2021). Tanshinone IIA Downregulates Lipogenic Gene Expression and Attenuates Lipid Accumulation through the Modulation of LXRα/SREBP1 Pathway in HepG2 Cells. Biomedicines.

[48] Gao M., Bu L., Ma Y., Liu D. (2013). Concurrent activation of liver X receptor and peroxisome proliferator-activated receptor alpha exacerbates hepatic steatosis in high fat diet-induced obese mice. PLoS ONE.

[49] de Bruin R.G., Shiue L., Prins J., de Boer H.C., Singh A., Fagg W.S., van Gils J.M., Duijs J.M.G.J., Katzman S., Kraaijeveld A.O. (2016). Quaking promotes monocyte differentiation into pro-atherogenic macrophages by controlling pre-mRNA splicing and gene expression. Nat. Commun..

[50] Gnoni G.V., Rochira A., Leone A., Damiano F., Marsigliante S., Siculella L. (2012). 3,5,3′triiodo-L-thyronine induces SREBP-1 expression by non-genomic actions in human HEP G2 cells. J. Cell. Physiol..

[51] Ferré P., Foufelle F. (2010). Hepatic steatosis: A role for de novo lipogenesis and the transcription factor SREBP-1c. Diabetes. Obes. Metab..

[52] Almatrafi M.M., Vergara-Jimenez M., Murillo A.G., Norris G.H., Blesso C.N., Fernandez M.L. (2017). Moringa Leaves Prevent Hepatic Lipid Accumulation and Inflammation in Guinea Pigs by Reducing the Expression of Genes Involved in Lipid Metabolism. Int. J. Mol. Sci..

[53] Tang L.Y., Chen Y., Rui B.B., Hu C.M. (2016). Resveratrol ameliorates lipid accumulation in HepG2 cells, associated with down-regulation of lipin1 expression. Can. J. Physiol. Pharmacol..

[54] Fowler S.D., Greenspan P. (1985). Application of Nile red, a fluorescent hydrophobic probe, for the detection of neutral lipid deposits in tissue sections: Comparison with oil red O. J. Histochem. Cytochem. Off. J. Histochem. Soc..

[55] Hoang N.A., Richter F., Schubert M., Lorkowski S., Klotz L.-O., Steinbrenner H. (2019). Differential capability of metabolic substrates to promote hepatocellular lipid accumulation. Eur. J. Nutr..

[56] Minami Y., Hoshino A., Higuchi Y., Hamaguchi M., Kaneko Y., Kirita Y., Taminishi S., Taruno A., Fukui M., Arany Z. (2023). Liver lipophagy ameliorates nonalcoholic steatohepatitis through lysosomal lipid exocytosis. Nat. Commun..

[57] Belfort R., Harrison S.A., Brown K., Darland C., Finch J., Hardies J., Balas B., Gastaldelli A., Tio F., Pulcini J. (2006). A placebo-controlled trial of pioglitazone in subjects with nonalcoholic steatohepatitis. N. Engl. J. Med..

[58] Wang G.-L., Fu Y.-C., Xu W.-C., Feng Y.-Q., Fang S.-R., Zhou X.-H. (2009). Resveratrol inhibits the expression of SREBP1 in cell model of steatosis via Sirt1-FOXO1 signaling pathway. Biochem. Biophys. Res. Commun..

[59] Akao Y., Kuranaga Y., Heishima K., Sugito N., Morikawa K., Ito Y., Soga T., Ito T. (2022). Plant hvu-MIR168-3p enhances expression of glucose transporter 1 (SLC2A1) in human cells by silencing genes related to mitochondrial electron transport chain complex I. J. Nutr. Biochem..

[60] Chen T., Ma F., Peng Y., Sun R., Xi Q., Sun J., Zhang J., Zhang Y., Li M. (2022). Plant miR167e-5p promotes 3T3-L1 adipocyte adipogenesis by targeting β-catenin. Vitr. Cell Dev. Biol. Anim..

[61] Aquilano K., Ceci V., Gismondi A., De Stefano S., Iacovelli F., Faraonio R., Di Marco G., Poerio N., Minutolo A., Minopoli G. (2019). Adipocyte metabolism is improved by TNF receptor-targeting small RNAs identified from dried nuts. Commun. Biol..

[62] Minutolo A., Potestà M., Gismondi A., Pirrò S., Cirilli M., Gattabria F., Galgani A., Sessa L., Mattei M., Canini A. (2018). Olea europaea small RNA with functional homology to human miR34a in cross-kingdom interaction of anti-tumoral response. Sci. Rep..

[63] Roglia V., Potestà M., Minchella A., Bruno S.P., Bernardini R., Lettieri-Barbato D., Iacovelli F., Gismondi A., Aquilano K., Canini A. (2022). Exogenous miRNAs from Moringa oleifera Lam. recover a dysregulated lipid metabolism. Front. Mol. Biosci..

[64] Cavalieri D., Rizzetto L., Tocci N., Rivero D., Asquini E., Si-Ammour A., Bonechi E., Ballerini C., Viola R. (2016). Plant microRNAs as novel immunomodulatory agents. Sci. Rep..

[65] Marzano F., Caratozzolo M.F., Consiglio A., Licciulli F., Liuni S., Sbisà E., D’Elia D., Tullo A., Catalano D. (2020). Plant miRNAs Reduce Cancer Cell Proliferation by Targeting MALAT1 and NEAT1: A Beneficial Cross-Kingdom Interaction. Front. Genet..

[66] Zhang D., Lee H., Jin Y. (2020). Delivery of Functional Small RNAs via Extracellular Vesicles In Vitro and In Vivo. Methods Mol. Biol..

[67] Müller F.A., Sturla S.J. (2019). Human in vitro models of nonalcoholic fatty liver disease. Curr. Opin. Toxicol..

[68] Zhang B., Pan X., Cannon C.H., Cobb G.P., Anderson T.A. (2006). Conservation and divergence of plant microRNA genes. Plant J..

[69] Alptekin B., Akpinar B.A., Budak H. (2016). A Comprehensive Prescription for Plant miRNA Identification. Front. Plant Sci..

[70] Dai X., Zhuang Z., Zhao P.X. (2018). psRNATarget: A plant small RNA target analysis server (2017 release). Nucleic Acids Res..

[71] Carmona-Saez P., Chagoyen M., Tirado F., Carazo J.M., Pascual-Montano A. (2007). GENECODIS: A web-based tool for finding significant concurrent annotations in gene lists. Genome Biol..

[72] Rao X., Huang X., Zhou Z., Lin X. (2013). An improvement of the 2^(-delta delta CT) method for quantitative real-time polymerase chain reaction data analysis. Biostat. Bioinforma. Biomath..

