# Supplementary material for: Plant miR8126-3p and miR8126-5p Decrease Lipid Accumulation through Modulation of Metabolic Genes in a Human Hepatocyte Model That Mimics Steatosis
Source: Int J Mol Sci. 2024 Jan 31;25(3):1721. doi: 10.3390/ijms25031721 (PMC10855419; doi:10.3390/ijms25031721)
Supplement: Supplementary file 1 [file ijms-25-01721-s001.zip › ijms-2811244-supplementary.pdf]

Supplementary material

Supplementary Table S1. Gene ontology (GO) enrichment analyses of putative human target genes of plant miR8126-3p.

**Plant miR8126-3p putative target genes**			
**Biological Process**	**Relative enrichment**	**Adjusted p value**	**Genes**
Negative regulation of platelet aggregation (GO:0090331)	107.16	0.029	*ADAMTS18*
Inorganic cation transmembrane transport (GO:0098662)	58.45	0.039	*ATP1B4*
Intracellular mRNA localization (GO:0008298)	428.64	0.028	*CASC3*
Regulation of nuclear-transcribed mRNA catabolic process, nonsense-mediated decay (GO:2000622)	321.48	0.028	
mRNA transport (GO:0051028)	23.38	0.028	*CASC3, QKI*
Regulation of translation (GO:0006417)	17.15	0.029	
RNA splicing (GO:0008380)	8.38	0.048	
snRNA processing (GO:0016180)	98.92	0.030	*INTS8*
snRNA 3′-end processing (GO:0034472)	61.23	0.039	
Tyrosine metabolic process (GO:0006570)	321.48	0.028	*IYD*
Thyroid hormone metabolic process (GO:0042403)	128.59	0.029	
Inner cell mass cell fate commitment (GO:0001827)	642.96	0.028	*LATS1*
Regulation of ubiquitin-dependent protein catabolic process (GO:2000058)	321.48	0.028	
Sister chromatid segregation (GO:0000819)	428.64	0.028	
Inner cell mass cellular morphogenesis (GO:0001828)	642.96	0.028	
Regulation of intracellular estrogen receptor signaling pathway (GO:0033146)	128.59	0.029	
Cytoplasmic sequestering of protein (GO:0051220)	142.88	0.029	
Regulation of organ growth (GO:0046620)	142.88	0.029	
Mammary gland epithelial cell differentiation (GO:0060644)	91.85	0.032	
Hippo signaling (GO:0035329)	67.68	0.038	
Negative regulation of protein localization to nucleus (GO:1900181)	64.30	0.038	
Regulation of protein-containing complex assembly (GO:0043254)	58.45	0.039	
Regulation of transforming growth factor beta receptor signaling pathway (GO:0017015)	47.63	0.045	
Cellular biogenic amine metabolic process (GO:0006576)	428.64	0.028	*MAOA*
Catecholamine metabolic process (GO:0006584)	116.90	0.029	
Positive regulation of signal transduction (GO:0009967)	116.90	0.029	
Neurotransmitter catabolic process (GO:0042135)	142.88	0.029	
Dopamine catabolic process (GO:0042420)	128.59	0.029	
Negative regulation of glycolytic process (GO:0045820)	107.16	0.029	*NCOR1*
Locomotor rhythm (GO:0045475)	107.16	0.029	
Negative regulation of production of miRNAs involved in gene silencing by miRNA (GO:1903799)	116.90	0.029	
Negative regulation of fatty acid metabolic process (GO:0045922)	116.90	0.029	
Negative regulation of androgen receptor signaling pathway (GO:0060766)	85.73	0.032	
Negative regulation of JNK cascade (GO:0046329)	47.63	0.045	
Axon ensheathment (GO:0008366)	183.70	0.029	*QKI*
Regulation of mRNA splicing, via spliceosome (GO:0048024)	64.30	0.038	
Long-chain fatty acid biosynthetic process (GO:0042759)	64.30	0.038	
Cytidine to uridine editing (GO:0016554)	107.16	0.029	*RBM47*
Regulation of steroid metabolic process (GO:0019218)	428.64	0.028	*RORA*
T-helper 17 cell differentiation (GO:0072539)	321.48	0.028	

Cellular response to sterol (GO:0036315)	428.64	0.028	
cGMP metabolic process (GO:0046068)	428.64	0.028	
Positive regulation of circadian rhythm (GO:0042753)	128.59	0.029	
Cerebellar Purkinje cell differentiation (GO:0021702)	107.16	0.029	
Cerebellar granule cell precursor proliferation (GO:0021930)	214.32	0.029	
Regulation of macrophage activation (GO:0043030)	116.90	0.029	
Nitric oxide biosynthetic process (GO:0006809)	80.37	0.034	
Regulation of smoothened signaling pathway (GO:0008589)	55.91	0.040	
Regulation of glucose metabolic process (GO:0010906)	53.58	0.041	
Positive regulation of vascular endothelial growth factor production (GO:0010575)	42.86	0.048	
Rhythmic process (GO:0048511)	17.15	0.029	*RORA, NCOR1*
Muscle cell differentiation (GO:0042692)	128.59	0.013	*RORA, QKI*
Positive regulation of signal transduction by p53 class mediator (GO:1901798)	142.88	0.029	*RPL37*
Negative regulation of ubiquitin protein ligase activity (GO:1904667)	98.92	0.030	
Type B pancreatic cell proliferation (GO:0044342)	142.88	0.029	*SIDT2*
RNA transport (GO:0050658)	116.90	0.029	
Type B pancreatic cell development (GO:0003323)	85.73	0.032	
Regulation of insulin secretion involved in cellular response to glucose stimulus (GO:0061178)	71.44	0.037	

Putative targets were identified with TAPIR and psRNATarget (scoring schema V1 and V1) and the annotation "GO Biological Process" of GeneCodis was used to identify enriched pathways. The table shows biological processes of miR8126-3p putative target genes that reported an adjusted p value < 0.05.

A

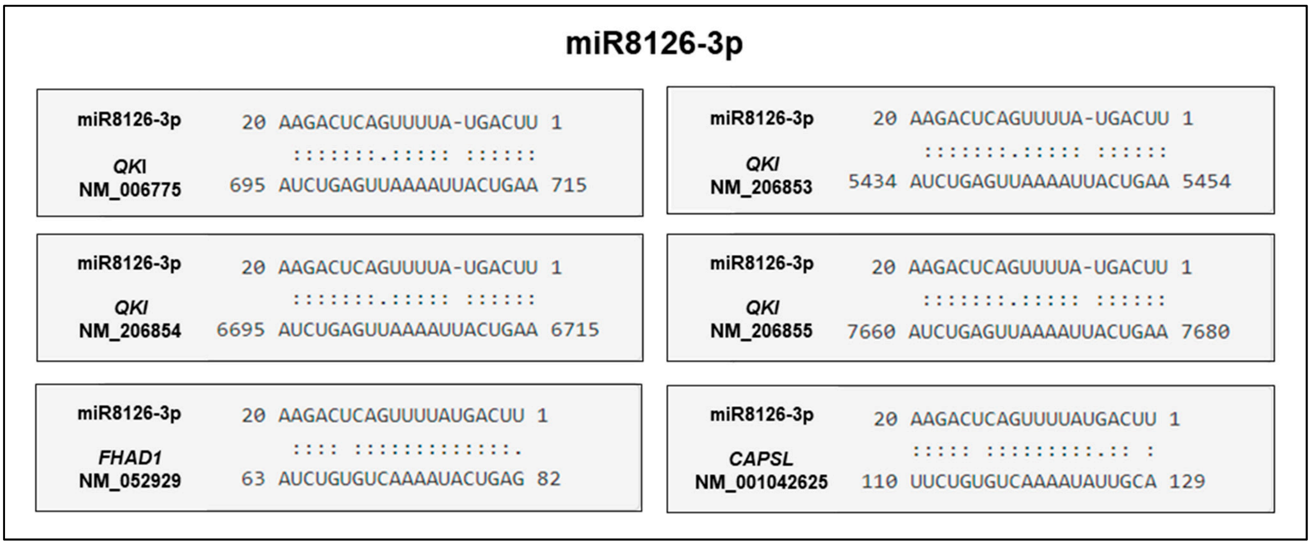

B

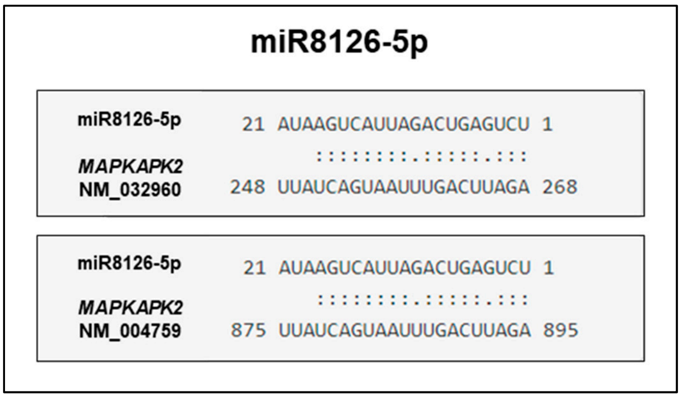

Supplementary Figure S1. ***In silico*** **prediction alignments between plant miR8126-3p and miR482c-5p and putative target transcripts. (A)** Alignments between plant miR8126-3p and the putative target transcripts of *QKI*, *FHAD1* and *CAPSL* genes. **(B)** Alignments between plant miR8126-5p and the putative target transcripts of *MAPKAPK2* gene. The predictions algorithms psRNATarget (scoring schema V1 and V2) and TAPIR were used to identify putative human target transcript of the mature sequence of plant miR8126-3p (5′-UUCAGUAUUUUGACUCAGAA-3′) and miR8126-5p (5′-UUCAGUAUUUUGACUCAGAA-3′) after the alignment with the cDNA library "Homo sapiens (human), transcript, Human genomic sequencing project" (from psRNATarget server). The Fig. represents alignments between miR8126-3p and miR8126-5p and common outputs to the prediction algorithms used. Abbreviations: *QKI* (Quaking Homolog, KH Domain RNA Binding) gene, *FHAD1* (Forkhead Associated Phosphopeptide Binding Domain 1) *CAPSL* (Calcyphosine Like), *MAPKAPK2* (MAPK Activated Protein Kinase 2, also named MK2).

Supplementary Table S2. Gene ontology (GO) enrichment analyses of putative human target genes of plant miR8126-5p.

Plant miR8126-5p putative target genes			
**Biological Process**	**Relative enrichment**	**Adjusted p value**	**Genes**
Histone exchange (GO:0043486)	125.02	0.042	*ANP32E*
Protein O-GlcNAcylation via threonine (GO:0097370)	1125.19	0.030	*EOGT*
Keratan sulfate catabolic process (GO:0042340)	562.59	0.036	*GNS*
Glycosaminoglycan metabolic process (GO:0030203)	102.29	0.042	
Glycosaminoglycan catabolic process (GO:0006027)	70.32	0.043	
Ubiquitin-dependent protein catabolic process (GO:0006511)	12.50	0.036	*KLHL8, NEDD4L, RNF14*
Protein ubiquitination (GO:0016567)	5.68	0.043	
p38MAPK cascade (GO:0038066)	93.77	0.042	*MAPKAPK2*
Macropinocytosis (GO:0044351)	140.65	0.042	
Regulation of tumor necrosis factor production (GO:0032680)	86.55	0.042	
Leukotriene metabolic process (GO:0006691)	66.19	0.043	
Regulation of cellular response to heat (GO:1900034)	70.32	0.043	
3′-UTR-mediated mRNA stabilization (GO:0070935)	56.26	0.046	
Toll-like receptor signaling pathway (GO:0002224)	48.92	0.046	
Regulation of interleukin-6 production (GO:0032675)	173.11	0.006	*MAPKAPK2, CXorf40A*
Positive regulation of caveolin-mediated endocytosis (GO:2001288)	375.06	0.036	*NEDD4L*
Negative regulation of sodium ion transmembrane transport (GO:1902306)	281.30	0.036	
Negative regulation of potassium ion transmembrane transport (GO:1901380)	102.29	0.042	
Negative regulation of sodium ion transmembrane transporter activity (GO:2000650)	112.52	0.042	
Regulation of membrane repolarization (GO:0060306)	86.55	0.042	
Negative regulation of potassium ion transmembrane transporter activity (GO:1901017)	160.74	0.042	
Regulation of membrane depolarization (GO:0003254)	112.52	0.042	
Negative regulation of protein localization to cell surface (GO:2000009)	93.77	0.042	
Response to metal ion (GO:0010038)	80.37	0.042	
Water homeostasis (GO:0030104)	187.53	0.042	
Regulation of potassium ion transmembrane transporter activity (GO:1901016)	187.53	0.042	
Ventricular cardiac muscle cell action potential (GO:0086005)	66.19	0.043	
Regulation of protein catabolic process (GO:0042176)	62.51	0.045	
Positive regulation of endocytosis (GO:0045807)	48.92	0.046	
Positive regulation of dendrite extension (GO:1903861)	48.92	0.046	
Cellular sodium ion homeostasis (GO:0006883)	51.14	0.046	
Regulation of dendrite morphogenesis (GO:0048814)	53.58	0.046	
Protein polyubiquitination (GO:0000209)	11.97	0.042	*NEDD4L, RNF14*
Ventricular cardiac muscle tissue development (GO:0003229)	281.30	0.036	*PPP1R13L*
Multicellular organismal homeostasis (GO:0048871)	375.06	0.036	
Hair cycle (GO:0042633)	80.37	0.042	
Embryonic camera-type eye development (GO:0031076)	86.55	0.042	
Cardiac right ventricle morphogenesis (GO:0003215)	80.37	0.042	
Regulation of androgen receptor signaling pathway (GO:0060765)	86.55	0.042	*RNF14*
Androgen receptor signaling pathway (GO:0030521)	56.26	0.046	
Sucrose catabolic process (GO:0005987)	1125.19	0.030	*SI*
Polysaccharide digestion (GO:0044245)	281.30	0.036	
Regulation of mitochondrial fission (GO:0090140)	140.65	0.042	*TMEM135*

Response to food (GO:0032094)	48.92	0.046
Peroxisome organization (GO:0007031)	46.88	0.047

Putative targets were identified with TAPIR and psRNATarget (scoring schema V1 and V1) and the annotation "GO Biological Process" of GeneCodis was used to identify enriched pathways. The table shows biological processes of miR8126-3p putative target genes that reported an adjusted p value < 0.05.

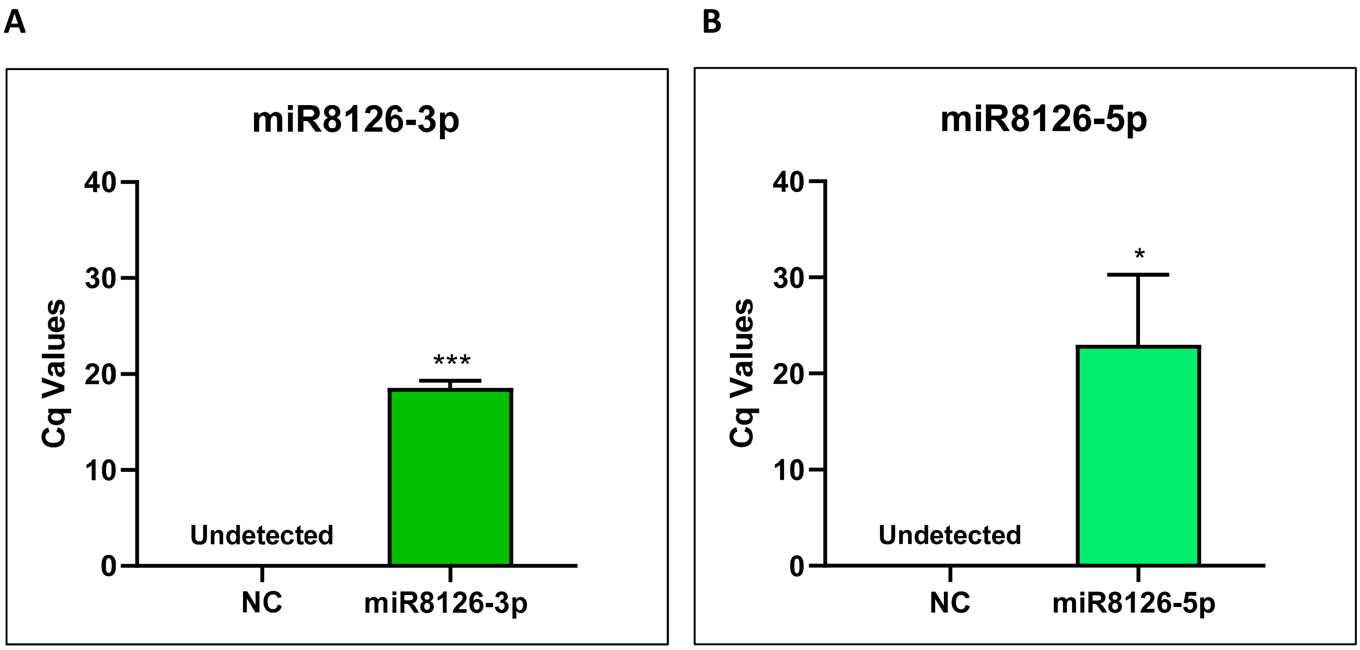

Supplementary Figure S2. Expression levels of plant miRNAs (A) miR8126-3p and (B) miR8126-5p in HepG2 cells after mimic transfection. HepG2 cells were transfected for 6 h with 50 nM of mirVana® mimics miR8126-3p (5′-UUCAGUAUUUUGACUCAGAA-3′), miR8126-5p (5′-UCUGAGUCAGAUUACUGAAUA-3′) and a scramble sequence as a control (Negative Control #1). miRNA expression levels were evaluated by qPCR. Results are Cq values ± standard error of the mean (SEM) (n= 2-3). Significance, determined with Student T-test (two-tailed), refers to the comparison of each plant miRNA mimic respect to the control: * $p<0.05$; *** $p<0.001$. Abbreviations: NC (negative control).

A

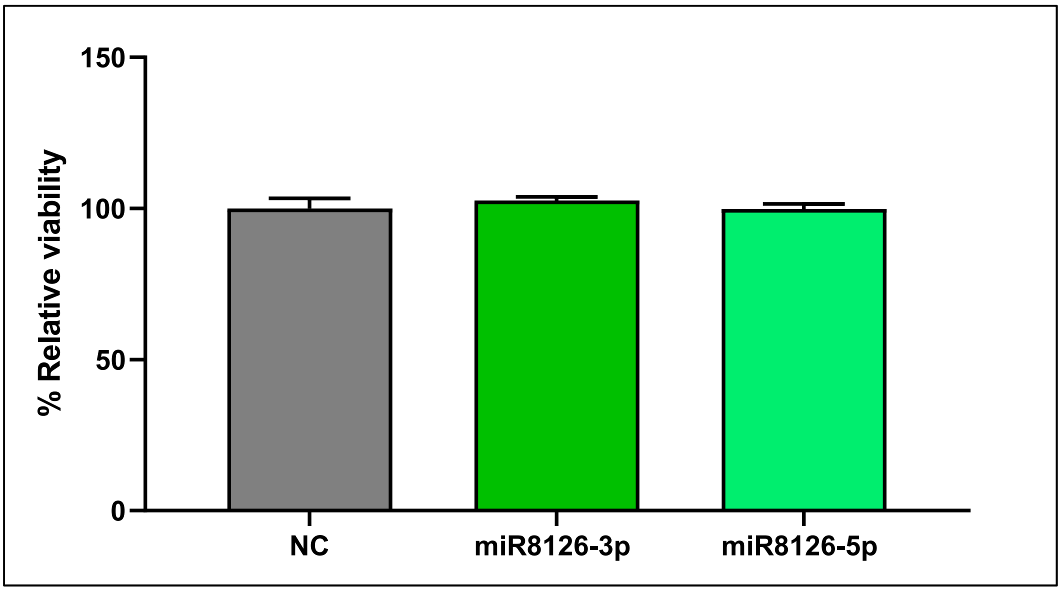

B

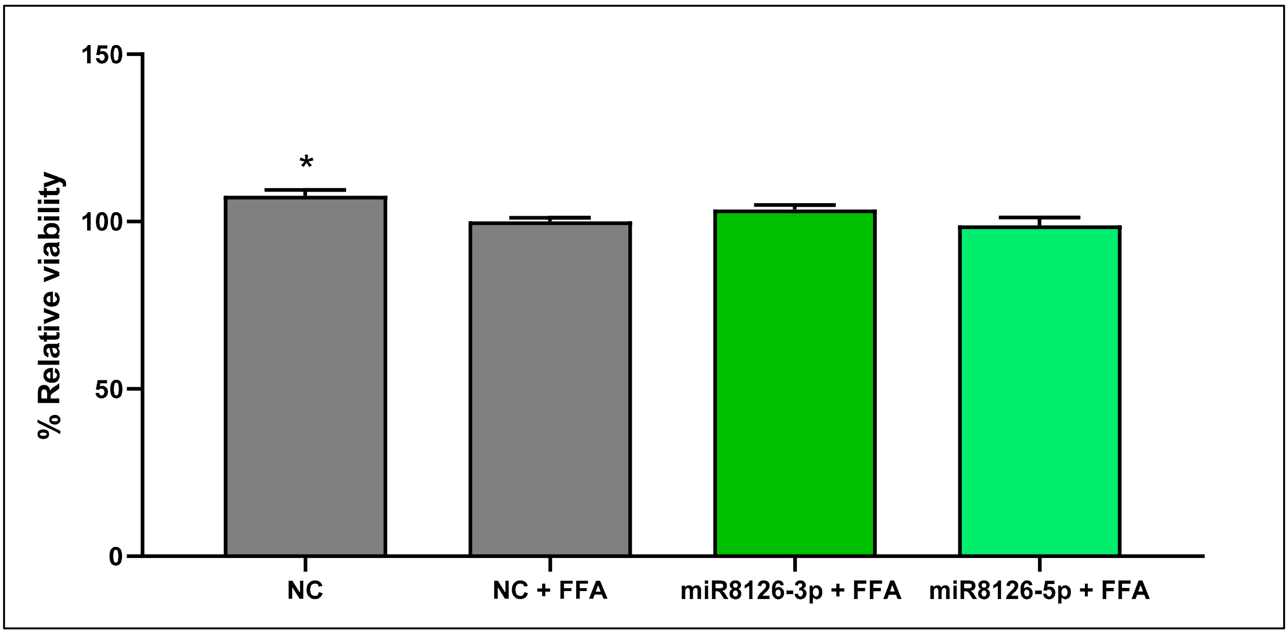

Supplementary Figure S3. Evaluation of the effect of plant miRNA mimics miR8126-3p and miR8126-5p on the viability of HepG2 cells. (A) Relative viability of HepG2 cells transfected with miR8126-3p and miR8126-5p. **(B)** Relative viability of HepG2 transfected with miR8126-3p and miR8126-5p and treated with free fatty acids. HepG2 cells were transfected for 48 h with 50 nM of mirVana mimics miR8126-3p (5′-UUCAGUAUUUUGACUCAGAA-3′), miR8126-5p (5′-UCUGAGUCAGAUUACUGAAUA-3′) and a scramble sequence as a control (Negative Control #1). Additionally, 48 h after transfections cells were treated with 0.5 mM of free fatty acids (proportion oleic:palmitic acids 2:1) for 3 h until cell viability assessment. Cell viability was determined by MTS assay in control cells and cells transfected with plant miRNA mimics untreated and treated with fatty acids. For Supplementary figure S3A, results are presented as the % cell viability relative to the negative control ± standard error of the mean (SEM) (n= 4); and significance, determined with Student t-test (two-tailed), was assessed by establishing comparison between control cells and each plant miRNA mimic treatment. For Supplementary Figure S3B, results are presented as the % cell viability relative to the negative control treated with fatty acids) ± standard error of the mean (SEM) (n=3-4); and significance refers to the comparison of each plant miRNA mimic and untreated control cells respect to the control treated with fatty acids: * p<0.05. Abbreviations: NC (negative control), FFA (free fatty acids).

A

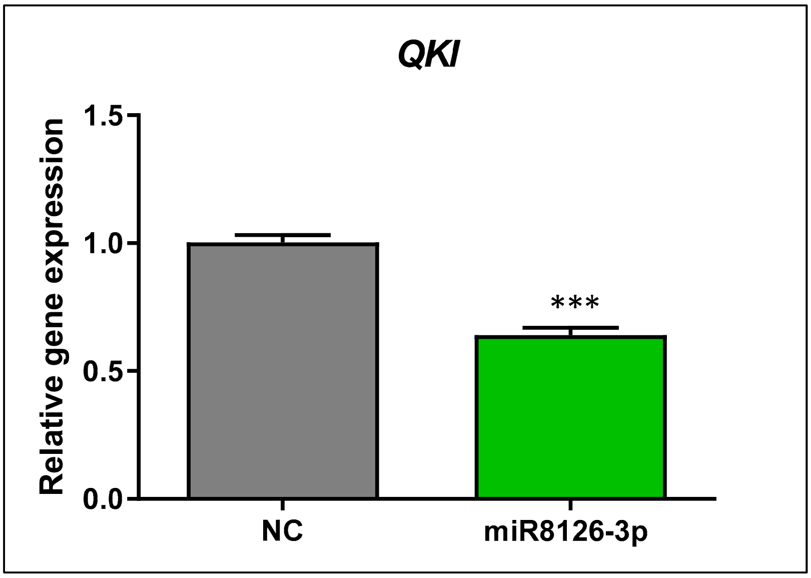

B

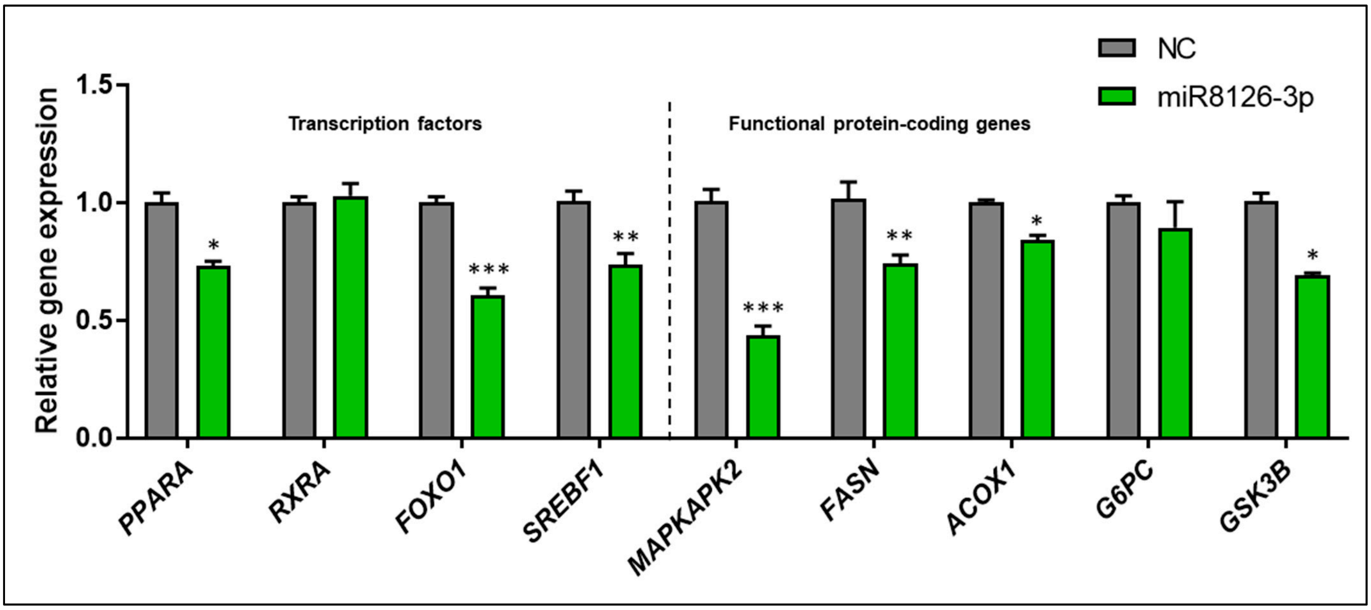

Supplementary Figure S4. Gene expression analyses of HepG2 cells transfected with plant miR8126-3p mimic. **(A)** mRNA levels of the putative target gene *QKI*. **(B)** mRNA levels of transcription factors (*PPARA*, *RXRA*, *FOXO1*, *SREBF1*) and functional protein coding genes (*MAPKAPK2, FASN*, *ACOX1*, *G6PC* and *GSK3B*) involved in glucose and lipid metabolism. HepG2 cells were transfected for 48 h with 50 nM of the mirVana mimic miR8126-3p (5′-UUCAGUAUUUUGACUCAGAA-3′) and a scramble sequence as a control (Negative Control #1). mRNA expression levels were quantified by qPCR. Cq values were normalized to the housekeeping gene *TBP* (TATA-box binding protein) and expressed as the gene expression levels relative to the negative control cells, calculated by the $2^{-\Delta\Delta Ct}$ method. Results are presented as the relative gene expression mean ± standard error of the mean (SEM) (n= 2-7). * p<0.05, ** p<0.01, *** p<0.001. Significance was determined applying Student T-test (two-tailed). Abbreviations: NC (negative control), *QKI* (Quaking Homolog, KH Domain RNA Binding), *MAPKAPK2* (MAPK Activated Protein Kinase 2), *PPARA* (Peroxisome Proliferator Activated Receptor Alpha), *RXRA* (Retinoid X Receptor Alpha), *FOXO1* (Forkhead Box O1), *SREBF1* (Sterol Regulatory Element Binding Transcription Factor 1), *FASN* (Fatty Acid Synthase), *ACOX 1* (Acyl-CoA Oxidase 1), *G6PC* (Glucose-6-Phosphatase Catalytic Subunit 1), *GSK3B* (Glycogen Synthase Kinase 3 Beta).

A

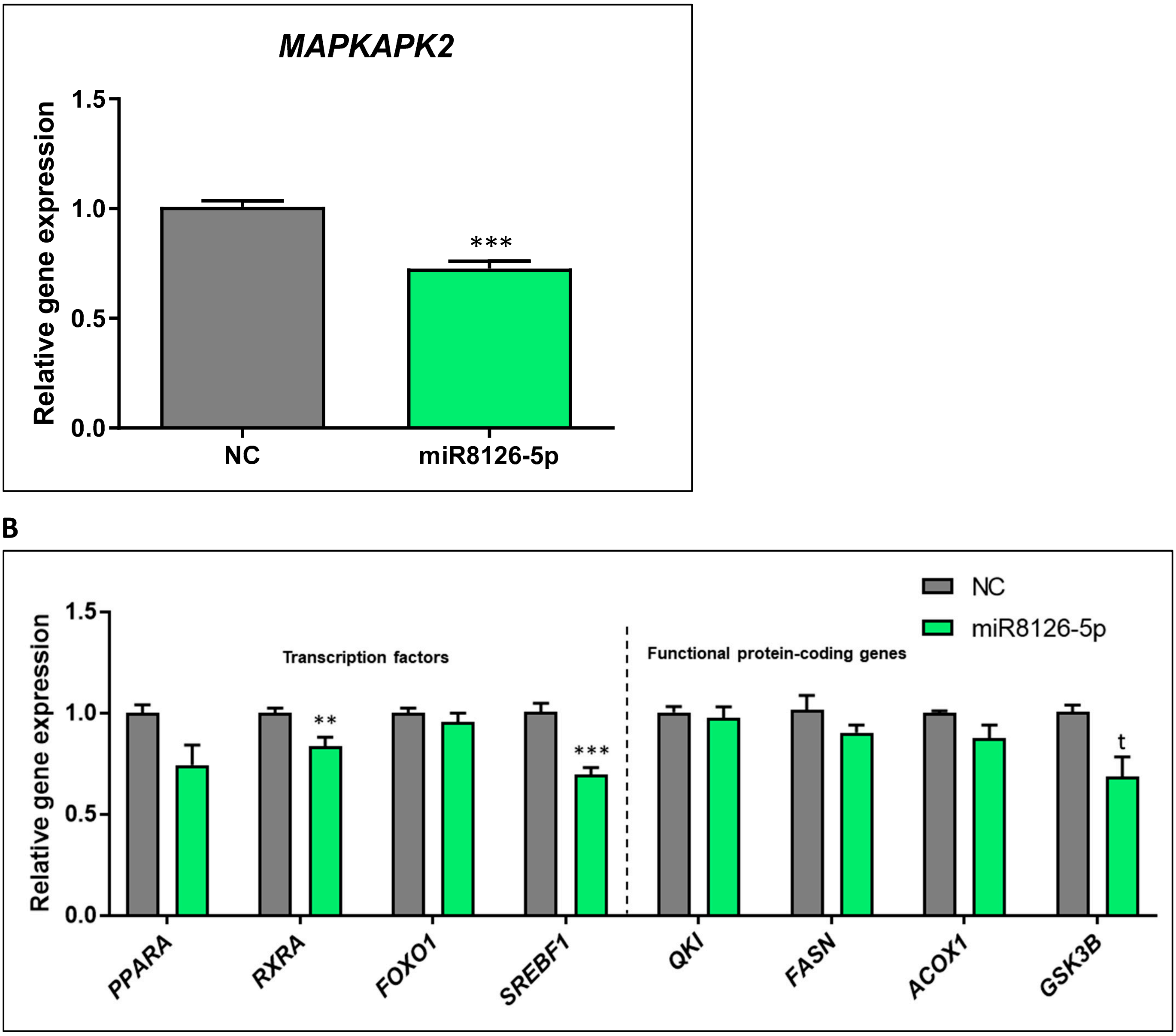

Supplementary Figure S5. Gene expression analyses of HepG2 cells transfected with plant miR8126-5p mimic. **(A)** mRNA levels of the putative target gene *MAPKAPK2*. **(B)** mRNA levels of transcription factors (*PPARA*, *RXRA*, *FOXO1*, *SREBF1*) and functional protein coding genes (*QKI, FASN*, *ACOX1*, *G6PC* and *GSK3B*) involved in glucose and lipid metabolism. HepG2 cells were transfected for 48 h with 50 nM of the mirVana mimic miR8126-5p (5′-UCUGAGUCAGAUUACUGAAUA-3′) and a scramble sequence as a control (Negative Control #1). mRNA expression levels were quantified by qPCR. Cq values were normalized to the housekeeping gene *TBP* (TATA-box binding protein) and expressed as the gene expression levels relative to the negative control cells, calculated by the $2^{-\Delta\Delta Ct}$ method. Results are presented as the relative gene expression mean ± standard error of the mean (SEM) (n= 2-7). ** $p<0.01$, *** $p<0.001$, t (tendency, GSK3B)= 0.0870. Significance was determined applying Student T-test (two-tailed). Abbreviations: NC (negative control), *MAPKAPK2* (MAPK Activated Protein Kinase 2), *QKI* (Quaking Homolog, KH Domain RNA Binding), *PPARA* (Peroxisome Proliferator Activated Receptor Alpha), *RXRA* (Retinoid X Receptor Alpha), *FOXO1* (Forkhead Box O1), *SREBF1* (Sterol Regulatory Element Binding Transcription Factor 1), *FASN* (Fatty Acid Synthase), *ACOX 1* (Acyl-CoA Oxidase 1), *G6PC* (Glucose-6-Phosphatase Catalytic Subunit 1), *GSK3B* (Glycogen Synthase Kinase 3 Beta).

Supplementary Table S3. Characteristics of HepG2 cells reverse transfection with mirVana™ miRNA mimics and Lipofectamine RNAiMAX Reagent (Thermo Fisher Scientific Inc.).

Assay	Plate type	Opti-MEM I + miRNA Mimic	Lipofectamine RNAiMAX	DMEM + 10% FBS	Cell number
Gene expression assays (miRNA and mRNA)	12-well	200 µL	2 µL	1,000 µL	100,000 cells
Cytotoxicity assays Red Nile staining	48-well	50 µL	0.5 µL (dilution 1:4 in Opti-MEM I to add 2 µL)	250 µL	25,000 cells
Triglyceride quantification assays	96-well	25 µL	0.25 µL (dilution 1:8 in Opti-MEM I to add 2 µL)	125 µL	12,500 cells

Volumes indicated correspond to one well.
